# Discovering multiple realistic TFBS motifs based on a generalized model

**DOI:** 10.1186/1471-2105-10-321

**Published:** 2009-10-07

**Authors:** Tak-Ming Chan, Gang Li, Kwong-Sak Leung, Kin-Hong Lee

**Affiliations:** 1 Department of Computer Science & Engineering, The Chinese University of Hong Kong, Shatin, N. T., Hong Kong

## Abstract

**Background:**

Identification of transcription factor binding sites (TFBSs) is a central problem in Bioinformatics on gene regulation. *de novo *motif discovery serves as a promising way to predict and better understand TFBSs for biological verifications. Real TFBSs of a motif may vary in their widths and their conservation degrees within a certain range. Deciding a single motif width by existing models may be biased and misleading. Additionally, multiple, possibly overlapping, candidate motifs are desired and necessary for biological verification in practice. However, current techniques either prohibit overlapping TFBSs or lack explicit control of different motifs.

**Results:**

We propose a new generalized model to tackle the motif widths by considering and evaluating a width range of interest simultaneously, which should better address the width uncertainty. Moreover, a meta-convergence framework for genetic algorithms (GAs), is proposed to provide multiple overlapping optimal motifs simultaneously in an effective and flexible way. Users can easily specify the difference amongst expected motif kinds via similarity test. Incorporating Genetic Algorithm with Local Filtering (GALF) for searching, the new GALF-G (G for generalized) algorithm is proposed based on the generalized model and meta-convergence framework.

**Conclusion:**

GALF-G was tested extensively on over 970 synthetic, real and benchmark datasets, and is usually better than the state-of-the-art methods. The range model shows an increase in sensitivity compared with the single-width ones, while providing competitive precisions on the *E. coli *benchmark. Effectiveness can be maintained even using a very small population, exhibiting very competitive efficiency. In discovering multiple overlapping motifs in a real liver-specific dataset, GALF-G outperforms MEME by up to 73% in overall *F-*scores. GALF-G also helps to discover an additional motif which has probably not been annotated in the dataset.

## Background

In this section, motif discovery is introduced, followed by the summarization of existing methods, and methods beyond the scope of this paper. Motivations are then given and the paper layout is presented.

### Motif Discovery

Transcription Factor Binding Sites (TFBSs) are small nucleotide fragments (usually ≤ 30 bp) in the cis-regulatory/intergenic regions in DNA sequences. Regulatory proteins, namely the Transcription Factors (TFs), bind in a sequence-specific manner to TFBSs to activate or suppress gene transcription (gene expression). Therefore, TFBSs are a critical component in gene regulation, and identification of TFBSs is a central problem for understanding gene regulation in molecular biology.

The DNA binding domain(s) of a TF can recognize and bind to a collections of similar TFBSs, from which a conserved pattern called motif can be obtained. Based on this phenomenon, *de novo *motif discovery using computational methods have been proposed to identify and predict TFBSs and their corresponding motifs. Motif discovery provides significant insights into the understanding of the mechanisms of gene regulation. It serves as an attractive alternative for providing pre-screening and prediction of unknown TFBS motifs to the expensive and laborious biological experiments such as DNA footprinting [1] and gel electrophoresis [2]. The recent technology of Chromatin immunoprecipitation (ChIP) [3,4] measures the binding of a particular TF to DNA using microarray technology at low resolution in a high-throughput manner, and produces more reliable input data of co-regulated genes for motif discovery [5].

### Existing Methods

#### Categorization

Because the conservation of motifs is often degenerated due to TFBS mutations, the searching is difficult (NP-hard [6]). Extensive algorithms have been proposed for *de novo *motif discovery since the last decades. There are two major representations for TFBS motifs (conserved patterns): (i) Consensus Representation and (ii) Matrix Representation; and there are two main different searching paradigms: (a) Enumeration Methods and (b) Stochastic Searching [4]. They are briefly described as follows:

**(i) Consensus Representation **is based on discrete strings. A simple model is to minimize the mismatches between the consensus and the TFBS instances [7-10].

**(ii) Matrix Representation **is usually a Position Frequency Matrix (PFM; see Table 1), or a Position Weight Matrix (PWM), to show the quantitative frequencies or weights of nucleotides in the motif. Representative evaluations for a motif matrix include Information Content (IC) [11], maximum a posterior (MAP) [12] and the Bayesian models [13] (see the probabilistic models in Methods section).

**Table 1:** Motif discovery example.

**Sequences *S***	**SIM *A***	**TFBSs *R***	**PFM Θ (4 × *w*(= 7))**
*S*_1_: acgtCGATTGCctaag	0000100000000000	CGATTGC	
*S*_2_: taTGATCGAtgacgca	0010000000000000	TGATCGA	A: 0.0 0.2 0.6 0.1 0.1 0.0 0.7
*S*_3_: cgaCAATTGAgcttac	0001000000000000	CAATTGA	C: 0.8 0.0 0.2 0.3 0.3 0.2 0.3
*S*_4_: gCGCTCGAcaagctgt	0100000000000000	CGCTCGA	G: 0.0 0.8 0.0 0.0 0.0 0.8 0.0
*S*_5_: cgttTGTCACAgtcta	0000100000000000	TGTCACA	T: 0.2 0.0 0.2 0.6 0.6 0.0 0.0
*S*_6_: tcagcCACACCCagct	0000010000000000	CACACCC	
*S*_7_: ccagagCGTCTGAttg	0000001000000000	CGTCTGA	Background: Θ_0_:
*S*_8_: gacttcaCGACTGAgc	0000000100000000	CGACTGA	*θ*_0*A *_= 0.24 *θ*_0*C *_= 0.29
*S*_9_: gctgcccatCGATTGA	0000000001000000	CGATTGA	*θ*_0*G *_= 0.24 *θ*_0*T *_= 0.23
*S*_10_: ccaggtacCGATTGCa	0000000010000000	CGATTGC	

An artificial example of motif discovery. It shows the sequences *S*, the SIM *A*, the motif instances *R*, the PFM Θ and the background frequencies Θ_0_. In sequences *S*, the nucleotides from the background are shown in lower case, while the nucleotides from the motif instances in upper case.

The searching techniques with respect to the two representations, are discussed below.

**(a) Enumeration Methods **are usually applied [7,8,14-16] to the consensus representation, but they do not scale up for long widths. However, they are useful to provide candidates for further searching and evaluations [5,17,18]. Weeder [15,16] is one well-known representative in this category.

**(b)Stochastic Searching **is usually applied to align TFBSs and obtain the motif matrix for the matrix representation. Typical techniques can be categorized into **local searching **[5,12] and **global searching**, where the latter can be classified into **(S) Single-point **and **(M) Multi-point or group-based searching**. Global searching is more likely to find the global optima compared with local searching. While Gibbs sampling is popular in motif discovery tools: e.g. BioProspector [19], AlignACE [20] and MotifSampler [21]). Its single-point nature requires numerous iterations to converge to the global optima, otherwise the performance may be affected significantly. Alternatively, the multi-point global searching approach, the genetic algorithm [22,23], has shown promising results in motif discovery [9,10,24-28]. There is great potential for them to be applied to more sophisticated models and provide multiple optimal motifs [26].

Table 2 summarizes the representations, the associated models and the searching techniques employed by the motif discovery methods. The table serves to illuminate the representative methods in each category including those we have compared in our experiments.

**Table 2:** Motif discovery methods summary.

**Representations** **(i) Consensus** **(ii) Matrix and Evaluations**		**(a) Enumerations**		**(b) Stochastic Search**		
		
		**Exhaustive**	**Non-exhaustive**	**Local**	**Global**	
					
					**Single-point (Gibbs Sampling)**	**Multi-point (GAs)**
(i)	Hamming	[7,8]	[14]	[17,18]		[9,10]
	
	*Z*-score		Weeder [15,16]			[24]

(ii)	IC		[41]		[46]	[10], GALF-P [28]
	
	Bayesian		BioOptimizer [40]		BioProspector [19] Motif Sampler [21]	GAME [27]
	
	MAP			MEME [12] MDScan [5]	AlignACE [20]	

Summary of the representative motif discovery methods. The methods included in our comparison experiments are shown with their names. IC stands for Information Content.

#### Methods beyond

Methods out of the scope of this paper but worth introducing are briefly mentioned as follows: Ensembles of multiple motif discovery programs have been recently shown to improve their performance [4,29,30]. However, modelling TFBS motifs is critically beneficial for better understanding and predicting novel motifs, and provides essential performance improvement for ensembles. As a result, we will focus on individual motif discovery methods in this paper.

Incorporating additional information sources [31,32] is another trend to improve the motif prediction accuracy. While extra requirements are needed for their success, the sequence-based motif discovery problem remains challenging [33-35] and calls for our serious attention because generalization and improvement on the sequence-based methods will without doubt help the integrated approaches.

### Motivations

#### Challenges

There still exist great challenges for *de novo *motif discovery algorithms to succeed. Challenges mainly include (i) NP hardness (ii), width uncertainty and (iii) multiple (overlapping) motifs, of which the latter two demand for more focus.

• **(i) NP hardness: **The most well-known challenge is the NP hardness [6] due to the unknown conservation degree, where extensive approaches have been proposed to achieve optimality under certain models, as surveyed in the last sub-sections.

• **(ii) Width uncertainty: **An often overlooked challenge in real-life problems is the uncertainty in the motif widths.

In real datasets, it is not easy to determine a single motif width (1) experimentally or (2) biologically. (1) Experimental: Annotated TFBSs are often affected by limited experimental resolutions, and it is thus difficult to choose any single width to fit the TFBSs before a motif can be discovered. (2) Biological: The most conserved binding contacts are between the short binding core of the target TFBS and the binding domain of a TF. The binding core may be fixed-width (< 6 bp). However, the short binding core may not provide enough binding affinity for its corresponding TF to recognize. Instead, a TF contain flexible segments of polypeptide chain, and these flexible arms work together with the DNA binding domain of the TF to add additional affinity [36]. The complication makes the effective width not easy to be fixed at a single value. For example, the TFBS widths vary in the familial binding cases of the Zn2-Cys6 motif [37].

**Figure 1:**
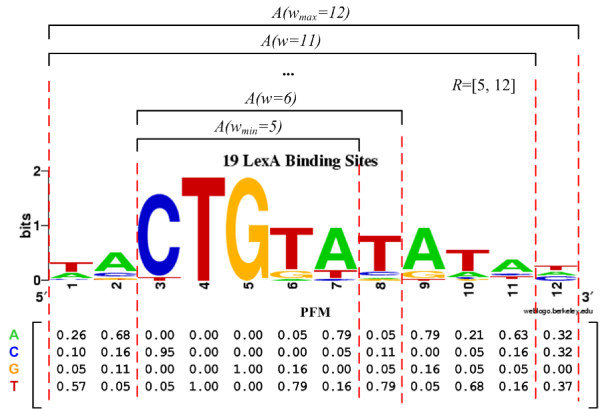
**The generalized model**. An example of the generalized model on the motif of 19 real LexA binding sites (the first 12 columns) from the SequenceLogo website. Each *A*(*w*_*i*_) is chosen based on the maximal *P*(*A*(*w*_*i*_)), where the bits bounded by the red dashes reflect *P*(*A*(*w*_*i*_)) for illustrative purpose. In practice, *P*(*A*(*w*_*i*_)) can be chosen flexibly.

Existing methods usually assume a known and fixed TFBS motif width or model a distribution around an expected width when there are uncertainties involved. The conservation contributed from different motif parts by varying the widths may be under-utilized in a single-width approach, and the so-called expected value may be misleading and biased. Statistical significance to rank different widths, e.g. E-value [38], is computational intensive and still only picks a single-value width at the end. In the illustrative example of a real motif with 19 LexA binding sites in Figure 1, if a single width is chosen, it may be 5 if only the stringent core part (3-7) is chosen; or it may be 12 if considering all columns (1-12). In the former case, the short motif may not be ranked higher than those non-TFBS frequent patterns happening by chance. In the latter case, since both highly and weakly conserved columns are evaluated equally, it may include additional false positives. On the contrary, modelling those uncertain bases with a range concept can better capture the different resolutions for assessing the motif signals, and thus potentially better describe the real TFBS motif.

• **(iii) Multiple (overlapping) motifs: **Another challenge which is not well handled is the overlapping nature of TFBSs for different motifs because competitive binding exists amongst different TFs in the same regulatory region. Current techniques used are mainly masking/erasing and implicit maintaining.

- Masking/erasing: These techniques can only discover one motif in a single execution, and thus several executions are required for outputting multiple motifs. Masking/erasing techniques also prohibits the subsequent discovery of the TFBSs overlapped with those previously masked ones. However, in real cases, different kinds of TFBSs may overlap with each other due to competitive binding of TFs.

- Implicit maintaining: There are existing methods to sample different motifs simultaneously but with little or no mechanism to explicitly distinguish different solutions or flexibly control the overlapping degrees of TFBSs. As a result, highly redundant motifs may be produced. If there are limited number of output solutions, redundant top-scored variant motifs will dominate and less-fit but different solutions will be missed. If non-redundant and different solutions need to be provided, a large output number has to be set and post-processing is required [39] with additional costs.

Therefore, it is desirable to discover multiple motifs more effectively and efficiently with certain flexible and explicit overlapping control.

#### Paper Layout

To overcome all these drawbacks of the existing de novo motif discovery algorithms, we propose **the generalized model **which presents a new angle to handle the variable motif widths to better reflects the biological uncertainty. Then we present **the meta-convergence framework **to support multiple optimal solutions with flexible overlapping control using similarity tests. Based on the generalized model and the framework, a new algorithm called **GALF-G **is developed.

The rest of the paper is arranged as follows. The generalized model, the meta-convergence framework and the new algorithm GALF-G are first given. Extensive experimental results are reported, including single/multiple motif discovery problems with fixed-width/variable widths inputs. A large number of both synthetic and real benchmark datasets are used in the experiments. After the substantial analysis of the results, discussion and conclusive remarks are made. The detailed implementations of our algorithm are given in the last Methods section.

## Results

In this section, we present **the generalized model **and **the meta-convergence framework **in detail, and propose the resulting **GALF-G **algorithm.

### The Generalized Motif Model

To tackle the challenge raised from the uncertainty of motif widths, we propose a new generalized model by considering a width range of interest simultaneously. A range is more practical and suitable for real biological cases for two reasons:

• First, it is easier to define a rough range than a particular width. All widths within contribute accordingly to the motif solution, and thus it is less sensitive than a wrongly chosen single width.

• Second, TFBSs of a motif in reality vary in their widths and exhibit certain higher degrees of conservation compared to the non-site fragments (the background). A range model can more appropriately capture the different conservation degrees than any single width.

Assume the width input is *R *= [*w*_*min*_, *w*_*max*_] and |R| = *w*_*max *_- *w*_*min *_+ 1, and a candidate solution, i.e. a set of TFBSs to form a motif, is defined as *A*, with the TFBS positions denoted by {*p*_*i*_}. The formal problem denotations and formulations are shown in the Methods section: The Proposed Model and Evaluation. The generalized model evaluates *A *based on the whole range *R*. An illustrative example is shown in Figure 1. The model or scoring function (illustrated by the heights of color nucleotides in the figure) for a fixed width *w*_*i *_is well established, e.g. a probabilistic model, denoted as *P *(*A*(*w*_*i*_)|*w*_*i*_), where *P*(*A*(*w*_*i*_)) is a part from the complete candidate solution *A *with respect to *w*_*i*_. The generalized model can then be formulated by summing them together as

(1)

For the most common case when there is no prior knowledge on which width is more likely to happen, *w*_*i *_can take a uniform distribution, i.e. *P*(*w*_*i*_) = 1/|*R*| for each *w*_*i*_. On the other hand, any prior distribution such as the Poisson one used in Bayesian models [40] can be also adopted. For each *w*_*i*_-component where *w*_*min *_≤ *w*_*i *_<*w*_*max*_, there are more than one choice and we only pick the component *A*(*w*_*i*_) by *argmax*(*P*(*A*(*w*_*i*_)|*w*_*i*_)) (caps in Figure 1). The additional computational cost compared to a fixed width model is *O*(|*R*|^2^), which is feasible since motif ranges (width variations) are usually short (≤ 10 bp). The major difference of the generalized model from the previous ones is that all the widths from the input range *R *contribute to the solution score/fitness in the model, rather than choosing a certain single width by *argmax*(*P*(*A*(*w*_*i*_)|*w*_*i*_) *P *(*w*_*i*_)), which has the risk of bias on a certain single value. If only one width is input, the generalized model reduces to one of the existing fixed-width models.

Intuitively, the generalized model is a weighted sum of the probability of different widths from the range *R*. It is compatible with the existing probability models and is even applicable to non-probability models, as long as there is a consistent expression of *P*(*A*(*w*_*i*_)); here it refers to an evaluation function in general. We employ the fixed-width probabilistic model in our generalized model, which will be discussed in detail in the Methods section.

### The Meta-convergence Framework

For practitioners in molecular biology and medical research, it is desirable that multiple optimal candidate motifs can be provided concurrently for biological verification. Due to the limitations of masking/erasing and implicit maintaining, it is desired to explicitly maintain different solutions with flexible (typically overlapping) control efficiently. To address these issues, we propose a meta-convergence framework employing Genetic Algorithm (GA) with the similarity test as the overlapping control.

**(i) The similarity test **is first introduced to fulfill flexible overlapping control over different motifs. Positional information is considered since the generalized model involves a width range *R *of positions. In particular, to compare two candidate solutions/individuals *A*_*a *_and *A*_*b*_, the test calculates the relaxed Hamming distance *h *between each pair of their aligned TFBS positions: (*A*_*a*_) and (*A*_*b*_) in sequence *i*,

(2)

where *tol *is the shift tolerance. The similarity test is passed, if

(3)

, where *dr *is defined as the difference ratio, *m *indicates the number of sequences, and *st *is the similarity threshold. When *dr *<*st*, *A*_*a *_and *A*_*b *_are considered to be similar, i.e. belong to the same motif kind. The intuitive settings of *tol, st *for different purposes, and how the test is applied are detailed and included in Methods: Meta-convergence Framework Details.

The similarity test proposed allows users to control the differences between the expected motifs in an easy and intuitive way. On the contrary, the other possible comparisons based on the PFM involve complicated cut-off which is not trivial to specify and counterintuitive for common users.

**(ii) Meta-convergence**, with the similarity test, monitors the convergence of different optimal solutions and adaptively controls the numbers of GA runs rather than using a relatively large fixed number of GA runs in previous works [27,28]. Furthermore, only a small number of candidates are subject to the similarity test to compete for the multiple optimal motifs, compared with the other method [26] that compares the whole population of solutions with non-trivial overhead. Therefore, the efficiency can be significantly improved. More details can be found in Methods: Meta-convergence Framework Details.

### GALF-G

Incorporating Genetic Algorithm with Local Filtering (GALF) with the generalized model and the meta-convergence framework, GALF-G (G for generalized) is proposed to discover multiple optimal motifs with flexible overlapping control using the similarity test. To fit into the generalized model with range input, the operators in GALF are extended accordingly and detailed in the Methods section: GALF-G implementations.

In the following section, we will report the results of GALF-G tested on both synthetic and real benchmark datasets for various cases, namely fixed-width, variable width, for single motif [with single (*K *= 1) or multiple outputs (*K *> 1) for single motif] and multiple motifs (*K *> 1) discoveries.

### Experiments

In this section, The summary of the experiments is introduced, and then the experimental results are reported and analyzed in corresponding categories. Finally experiments concerning the efficiency of GALF-G are presented.

### Experiment Summary

First of all, the evaluation measurements are introduced here. For most experiments except the benchmark ones [34,35], the measurements employed are the site level (prefix *s*) ones: positive predictive value/precision *sPPV*, sensitivity/recall *sSn *and F-score *sF *with shift restrictions, similar to [27,28]. The advantage is that they reflect both site level and part of the nucleotide level performances concisely. For the benchmark experiments, we have to follow their standard measurements which employ looser site level measurements but introduce additional nucleotide level (prefix *n*) PPV (*nPPV*) and sensitivity (*nSn*), as well as performance coefficient (*PC*) [14,33-35] and correlation coefficient (*CC*) [33,35] on both levels [see Additional file 1 for details of evaluation measurements for different experiments].

**(i) Single motif discovery experiments (***K *= 1**) **were firstly performed to test the generalized model. GALF-G was verified on the 800 synthetic datasets from [28], and compared with other state-of-the-art algorithms with fixed-width inputs as a special/degenerative case. GALF-G was then further tested on the 8 real datasets employed in GAME [27] with both fixed-width (the assumed true widths from [27]) inputs and range (variable widths) inputs relatively close to the true widths. The challenges raised by the eukaryotic benchmark [33,35] are then addressed, where there is no dataset-specific prior knowledge on the motif widths and only single motif outputs (*K *= 1) and compared.

**(ii) Multiple motifs experiments (***K *> 1**) **were then performed for two scenarios. In the first scenario, since multiple candidates are desirable for biological testing even for single motif discovery [34], GALF-G was tested and compared with the state-of-the-art algorithms on the 62 *E. coli *benchmark datasets [34], without dataset-specific prior knowledge on the motif widths. In the second scenario, since it is also desirable to discover different real motifs simultaneously, GALF-G, GAME and MEME were tested on the real liver-specific dataset with multiple (overlapping) motifs. Investigating into the exceptional case of GAME's 8 datasets using GALF-G with multiple motifs discovery, we discovered a putative motif not annotated in the dataset previously has been identified.

### Single Fixed-width Motif Discovery on Synthetic Data

GALF-G was first verified in the special cases of fixed-width single motif discovery (*K *= 1) on the 800 synthetic datasets used to test GALF-P in [28], which had performed best for these fixed width cases. We compared GALF-G with GALF-P, GAME, MEME, BioProspector (BioPro.), and BioOptimizers based on MEME and BioProspector. Weeder was not compared because it cannot be run on the long-width (16) datasets due to its width limit of 12. Details on generating the datasets were provided in [28] [see Additional file 1]. The average *F*-scores *sF *on the site level for each scenario are presented in Table 3, with the best results shown in bold. The full table with precisions (*sPPV*), recalls (*sSn*), including BioOptimizer results (almost identical to MEME and BioProspector), is shown in [Additional file 1]. GALF-G and GALF-P are in general the best among all scenarios, especially in the difficult scenarios (for example, short widths and low conservation). GALF-G is slightly better than GALF-P in the last 4 scenarios. To compare GALF-G with another close competitor, MEME, t-test was employed [see Additional file 1]. GALF-G is shown to be better than MEME within the significance level 0.05 in 4 out of the 6 scenarios with better *sF*, while MEME shows no convincing significance of being better in the other 2 scenarios.

**Table 3:** Synthetic experiments.

**Scenarios**	**GALF-G**	**GALF-P**	**GAME**	**MEME**	**BioPro.**
**Width/Num/Con**					
	
Short/Small/Low	**0.48 **± 0.29	0.44 ± 0.27	0.30 ± 0.30	0.39 ± 0.35	0.39 ± 0.31
Short/Large/Low	**0.55 **± 0.22	**0.55 **± 0.22	0.36 ± 0.30	0.42 ± 0.29	0.45 ± 0.23
Long/Small/Low	**0.89 **± 0.13	**0.89 **± 0.14	0.82 ± 0.22	0.88 ± 0.14	0.83 ± 0.14
Long/Large/Low	**0.91 **± 0.06	**0.91 **± 0.05	0.90 ± 0.07	0.90 ± 0.07	0.80 ± 0.11
Short/Small/High	0.84 ± 0.07	0.80 ± 0.09	0.75 ± 0.23	**0.85 **± 0.07	0.78 ± 0.12
Short/Large/High	**0.85 **± 0.04	0.83 ± 0.05	0.83 ± 0.10	0.83 ± 0.04	0.76 ± 0.06
Long/Small/High	**0.98 **± 0.02	**0.98 **± 0.03	0.97 ± 0.03	**0.98 **± 0.02	0.97 ± 0.03
Long/Large/High	**0.99 **± 0.01	0.97 ± 0.02	0.98 ± 0.01	0.98 ± 0.01	0.96 ± 0.02
	
Average	**0.81**	0.80	0.74	0.78	0.74

Average site level *F*-scores for the 800 fixed-width synthetic datasets experiments. ± indicates the standard deviation (over the 100 datasets generated for each scenario). Width: the motif width, Num: the number of sequences and Con: conservation degree.

We do not expect great differences between GALF-G and other algorithms here, because under the fixed-width cases the generalized model is similar to other models in representative power. The experiments demonstrate the search capability of GALF-G is comparable to or better than the previous best GALF-P on the synthetic datasets. The main reason is that they use similar effective searching techniques based on local filtering [28]. The results from the synthetic data can be interpreted intuitively with respect to searching difficulties, because their respective conservation degrees are explicitly generated. For variable-width (range) cases, the complicated nature of different conservation degrees of TFBSs is not easy to model or evaluate with synthetic data, hence it is more appropriate to test different methods with substantial real datasets, and the experimental results are presented in the following sub-sections.

### Single Motif Discovery on Real Datasets

In this sub-section, GALF-G was evaluated and compared with other methods on the 8 real datasets used to test GAME [27], for both fixed and variable widths cases in single motif discovery (*K *= 1).

Information of the 8 datasets is shown in Table 4. The CRP dataset contains the binding sites for cyclic AMP receptor, and has been widely tested since [41] was published. The ERE dataset contains the binding sites for the ligand-activated enhancer protein estrogen receptor (ER) [42]. The E2F datsets correspond to TFBSs of the E2F family from mammalian sequences [43]. CREB, MEF2, MyoD, SRF and TBP are chosen from the ABS database of annotated regulatory binding sites [44]. More details of the datasets can be found in [27].

**Table 4:** The 8 real datasets.

	**CREB**	**CRP**	**ERE**	**E2F**	**MEF2**	**MyoD**	**SRF**	**TBP**
	
*N*	17	18	25	25	17	17	20	95
*l*	200	105	200	200	200	200	200	200
*w*_*true*_	8	22	13	11	7	6	10	6
#_*t*_	19	23	25	27	17	21	36	95

Summary of the 8 real datasets. *N *is the number of sequences, *l *is the sequence length, *w*_*true *_is the motif width, and #_*t *_is the number of TFBSs embedded.

The comparison studies for fixed and variable widths cases are given as follows:

**(i) Fixed-width single motif discovery (***K *= 1**) **experiments were performed, where GALF-P was previously tested and compared with GAME in a fixed-width manner. GALF-G shows comparable overall *F*-scores *sF *(0.81) to the best average results from GALF-P (0.82) and is better than GAME (0.61) by 33% on average from 20 runs. While GALF-P shows significantly smaller variations than GAME in the performance [28], GALF-G shows even more stable and robust performance than GALF-P, which is discussed further in the Efficiency Experiments.

We have also tried Weeder [15,16] on part of the datasets because Weeder can only handle widths 6, 8, 10 and 12. Weeder is optimized for several width range modes [16] rather than fixed widths and will be formally compared in the following range experiments. For the fixed-width experiments, only CREB, MyoD, SRF and TBP were tested. The averaged *sPPV*, *sSn *and *sF *of Weeder for the 4 datasets are 0.43, 0.63 and 0.51, respectively. On the other hand, GALF-G is better where the corresponding values are 0.79, 0.83 and 0.81.

Similar to the conclusion on fixed-width synthetic experiments, GALF-G demonstrates competitive searching capacity on the fixed-width real data experiments, while GALF-G makes a looser assumption.

**(ii) (K = 1) variable-width (range) experiments **were performed, where GALF-G was compared with GAME, MEME, Weeder, and FlexModule from CisGenome [45] on the previous 8 real datasets. The additional FlexModule is a Gibbs sampling [46] motif discovery module implemented in the recent integrated system CisGenome [45] for analyzing transcriptional regulation.

For each dataset, 3 different width ranges were input for testing where

(4)

Each range represented variations of ± 3 bp on the width *w*_*i *_while the lower bound for *w*_*min*((*i*) _was set to 5 because it is rare for a motif width being smaller than 5. With increasing *i*, *w*_*i *_= *w*_*true *_+ (*i *- 1) reflects larger divergence of shift from the biological truth *w*_*true *_[See Additional file 1 for the running parameters]. The average results of executing each program 20 times are shown in Tables 5 and 6. Weeder is deterministic, and MEME performs constantly in different runs for a same dataset (as contrast to different datasets in Table 3), so there are no standard deviations shown for them.

**Table 5:** Experiments on the 8 real datasets (1).

**Datasets**	**GALF-G**			**GAME**		
	**sPPV**	**sSn**	**sF**	**sPPV**	**sSn**	**sF**
		
CREB						
*R*_1_	0.76 ± 0.00	0.68 ± 0.00	**0.72 **± 0.00	0.34 ± 0.37	0.35 ± 0.36	0.34 ± 0.36
*R*_2_	0.75 ± 0.06	0.68 ± 0.04	**0.71 **± 0.05	0.33 ± 0.34	0.34 ± 0.35	0.33 ± 0.34
*R*_3_	0.76 ± 0.00	0.68 ± 0.00	**0.72 **± 0.00	0.39 ± 0.36	0.38 ± 0.35	0.38 ± 0.35
		
CRP						
*R*_1_	0.94 ± 0.00	0.73 ± 0.02	**0.82 **± 0.01	0.79 ± 0.02	0.78 ± 0.00	0.78 ± 0.01
*R*_2_	0.89 ± 0.02	0.74 ± 0.00	**0.81 **± 0.01	0.82 ± 0.00	0.78 ± 0.00	0.80 ± 0.00
*R*_3_	0.79 ± 0.06	0.71 ± 0.04	0.75 ± 0.05	0.93 ± 0.03	0.66 ± 0.03	0.77 ± 0.01
		
ERE						
*R*_1_	0.64 ± 0.02	0.83 ± 0.02	0.72 ± 0.02	0.53 ± 0.00	0.80 ± 0.00	0.63 ± 0.00
*R*_2_	0.67 ± 0.03	0.85 ± 0.03	0.75 ± 0.03	0.55 ± 0.04	0.79 ± 0.02	0.65 ± 0.02
*R*_3_	0.77 ± 0.05	0.84 ± 0.01	**0.80 **± 0.03	0.60 ± 0.04	0.80 ± 0.03	0.69 ± 0.03
		
E2F						
*R*_1_	0.79 ± 0.02	0.84 ± 0.03	**0.81 **± 0.02	0.76 ± 0.09	0.84 ± 0.10	0.80 ± 0.10
*R*_2_	0.79 ± 0.00	0.81 ± 0.00	**0.80 **± 0.00	0.72 ± 0.00	0.85 ± 0.00	0.78 ± 0.00
*R*_3_	0.79 ± 0.00	0.81 ± 0.00	**0.80 **± 0.00	0.75 ± 0.00	0.78 ± 0.00	0.76 ± 0.00
		
MEF2						
*R*_1_	0.93 ± 0.00	0.82 ± 0.00	**0.88 **± 0.00	0.65 ± 0.29	0.75 ± 0.33	0.69 ± 0.30
*R*_2_	0.94 ± 0.00	1.00 ± 0.00	**0.97 **± 0.00	0.73 ± 0.26	0.77 ± 0.28	0.75 ± 0.27
*R*_3_	1.00 ± 0.00	1.00 ± 0.00	**1.00 **± 0.00	0.93 ± 0.00	0.83 ± 0.03	0.88 ± 0.01
		
MyoD						
*R*_1_	0.33 ± 0.04	0.42 ± 0.05	**0.37 **± 0.04	0.13 ± 0.10	0.16 ± 0.10	0.14 ± 0.10
*R*_2_	0.21 ± 0.01	0.23 ± 0.02	**0.21 **± 0.05	0.12 ± 0.11	0.16 ± 0.16	0.11 ± 0.11
*R*_3_	0.25 ± 0.00	0.29 ± 0.00	**0.25 **± 0.06	0.13 ± 0.12	0.14 ± 0.15	0.13 ± 0.14
		
SRF						
*R*_1_	0.72 ± 0.04	0.87 ± 0.03	**0.79 **± 0.03	0.71 ± 0.02	0.87 ± 0.04	0.78 ± 0.03
*R*_2_	0.74 ± 0.03	0.78 ± 0.04	0.76 ± 0.03	0.66 ± 0.02	0.87 ± 0.01	0.75 ± 0.02
*R*_3_	0.70 ± 0.02	0.74 ± 0.08	0.72 ± 0.05	0.70 ± 0.06	0.77 ± 0.05	0.73 ± 0.02
		
TBP						
*R*_1_	0.86 ± 0.01	0.82 ± 0.02	**0.84 **± 0.01	0.80 ± 0.08	0.75 ± 0.12	0.77 ± 0.09
*R*_2_	0.87 ± 0.02	0.86 ± 0.02	**0.87 **± 0.01	0.79 ± 0.05	0.78 ± 0.04	0.78 ± 0.03
*R*_3_	0.87 ± 0.02	0.86 ± 0.02	**0.86 **± 0.02	0.71 ± 0.17	0.74 ± 0.18	0.72 ± 0.18

Average	0.74	0.75	**0.74**	0.61	0.66	0.62

Average results (precision (*sPPV*), recall (*sSn*) and *F*-scores (*sF*) are averaged separately) of GALF-G and GAME on the 8 datasets. Each range *R*_*i *_= [*w *+ (*i *- 1) - 3, *w *+ (*i *- 1) + 3] in general indicates different shifts *i *from the true width *w*. ± shows the standard deviation (based on 20 independent runs of each dataset with each range). The results with best *sF *among this table and Table 6 are shown in bold.

**Table 6:** Experiments on the 8 real datasets (2).

**Datasets**	**MEME**			**Weeder**			**FlexModule**		
	**sPPV**	**sSn**	**sF**	**sPPV**	**sSn**	**sF**	**sPPV**	**sSn**	**sF**
			
CREB					medium				
*R*_1_	0.73	0.58	0.65	0.44	0.84	0.58	0.68 ± 0.04	0.76 ± 0.04	0.72 ± 0.04
*R*_2_	0.83	0.53	0.65	0.44	0.84	0.58	0.62 ± 0.22	0.69 ± 0.24	0.65 ± 0.23
*R*_3_	0.83	0.53	0.65	0.44	0.84	0.58	0.67 ± 0.07	0.72 ± 0.07	0.69 ± 0.07
			
CRP					large				
*R*_1_	0.93	0.61	0.74	0.41	0.71	0.52	0.94 ± 0.14	0.55 ± 0.11	0.69 ± 0.12
*R*_2_	0.89	0.70	0.78	0.41	0.71	0.52	0.97 ± 0.07	0.56 ± 0.06	0.70 ± 0.06
*R*_3_	0.89	0.70	**0.78**	0.41	0.71	0.52	0.96 ± 0.13	0.50 ± 0.10	0.65 ± 0.11
			
ERE					large				
*R*_1_	0.88	0.60	0.71	0.29	0.64	0.40	0.74 ± 0.03	0.85 ± 0.01	**0.79 **± 0.02
*R*_2_	0.88	0.60	0.71	0.29	0.64	0.40	0.73 ± 0.02	0.85 ± 0.02	**0.79 **± 0.02
*R*_3_	0.88	0.60	0.71	0.29	0.64	0.40	0.68 ± 0.17	0.77 ± 0.24	0.72 ± 0.21
			
E2F					large				
*R*_1_	0.78	0.67	0.72	0.23	0.93	0.37	0.56 ± 0.28	0.58 ± 0.29	0.57 ± 0.28
*R*_2_	0.83	0.70	0.76	0.23	0.93	0.37	0.60 ± 0.29	0.60 ± 0.29	0.60 ± 0.29
*R*_3_	0.78	0.67	0.72	0.23	0.93	0.37	0.63 ± 0.25	0.62 ± 0.25	0.63 ± 0.25
			
MEF2					medium				
*R*_1_	0.93	0.82	0.88	0.01	0.06	0.02	0.86 ± 0.02	1.00 ± 0.00	**0.93 **± 0.01
*R*_2_	0.93	0.82	0.88	0.01	0.06	0.02	0.79 ± 0.27	0.90 ± 0.31	0.84 ± 0.29
*R*_3_	0.93	0.82	0.88	0.01	0.06	0.02	0.88 ± 0.02	0.99 ± 0.04	0.93 ± 0.02
			
MyoD					small				
*R*_1_	0.00	0.00	0.00	0.07	0.10	0.08	0.00 ± 0.00	0.00 ± 0.00	0.00 ± 0.00
*R*_2_	0.00	0.00	0.00	0.07	0.10	0.08	0.00 ± 0.00	0.00 ± 0.00	0.00 ± 0.00
*R*_3_	0.00	0.00	0.00	0.07	0.10	0.08	0.00 ± 0.00	0.00 ± 0.00	0.00 ± 0.00
			
SRF					large				
*R*_1_	0.65	0.86	0.74	0.54	0.63	0.58	0.64 ± 0.00	0.87 ± 0.02	0.73 ± 0.01
*R*_2_	0.70	0.86	**0.78**	0.54	0.63	0.58	0.63 ± 0.01	0.82 ± 0.05	0.71 ± 0.02
*R*_3_	0.70	0.86	**0.78**	0.54	0.63	0.58	0.64 ± 0.00	0.86 ± 0.01	0.74 ± 0.00
			
TBP					small				
*R*_1_	0.70	0.67	0.69	0.56	0.90	0.69	0.47 ± 0.32	0.59 ± 0.40	0.53 ± 0.35
*R*_2_	0.70	0.67	0.69	0.56	0.90	0.69	0.41 ± 0.34	0.51 ± 0.42	0.45 ± 0.38
*R*_3_	0.70	0.67	0.69	0.56	0.90	0.69	0.45 ± 0.34	0.55 ± 0.41	0.49 ± 0.37

Average	0.71	0.61	0.65	0.32	0.60	0.40	0.61	0.63	0.61

Average results of MEME, Weeder and FlexModule in the same comparison experiments described in Table 5. Weeder was run with the width mode (small: 6, 8; medium: 6, 8, 10; large 6, 8, 10, 12) that are closest to the ranges *R *for each dataset.

In most cases (19/24) GALF-G achieves the best *F*-scores *sF *on the site level, as well as the average *sPPV*, *sSn *and *sF *averaged on all the cases. The overall *F*-score of GALF-G is 19% better than GAME, 14% better than MEME, 85% better than Weeder, and 21% better than FlexModule. The standard deviations of GALF-G are also lower than GAME and FlexModule in most cases. The t-test on *sF *shows that GALF-G is better than MEME in 20 cases within significance level 0.01, and in 1 case within significance level 0.02, while MEME is better in 3 cases within level 0.01. It should be noted that GALF-G significantly outperforms the other algorithms in *sSn*, probably because the generalized model not only predicts motifs as precise as the other models, but also accepts more correct TFBSs based on a wider range than single widths.

The above experiments demonstrate that with a range relatively close to the true widths, GALF-G with the generalized model shows favorable performance even compared with the results based on E-values. In fact, the performance with the input width ranges close to the true widths is comparable to that with fixed-width inputs, except for the MyoD dataset. The exceptional case of MyoD will be investigated separately and shown containing multiple motifs later.

To summarize, on the 8 real datasets for single motif discovery, GALF-G demonstrates competitive performance in fixed-width experiments, and provides obvious improvement over other methods in variable-width (range) experiments. For the cases without much prior information on the exact widths, experiments will be described in the next sub-sections.

### Single Motif Discovery Challenges on Eukaryotic Benchmarks

The recent well-known eukaryotic benchmark by Tompa et al [33] imposes great challenges to motif discovery algorithms. The problems of Tompa et al benchmark include the insufficient signals (few but long sequences) and inappropriate evaluation methods (unclear expert-tuned parameters for running and single top-scored motif outputs for comparisons) [See Additional file 1 for a more detailed discussion]. It has been indicated that many motifs in the Tompa et al benchmark are not able to be discriminated by common motif models from remaining sequence [35]. An improved benchmark [35] has thus been proposed for being more suitable to evaluate motif discovery algorithms. The algorithm benchmark suite [35] extracts motifs from TRANSFAC and includes representative eukaryotic species. There are 50 datasets with backgrounds generated by Markov models and 50 with real cis-regulatory region backgrounds. The widths are not given in the benchmark and thus a uniform width range input has to be set for all experiments. The additional evaluation measure corresponding to this benchmark is the nucleotide level correlation coefficient (*nCC*) [33-35].

GALF-G was tested on the corresponding algorithm benchmark suite [35] and compared with MEME and Weeder, the two most widely used algorithms [see Additional file 1 for the running parameters of GALF-G]. The average results of *nSn*, *nPPV*, *nPC *and *nCC *are shown in Table 7. For Markov backgrounds, GALF-G is 31% better than MEME, 214% than Weeder in *nPC*, and 42% better than MEME, 165% than Weeder in *nCC*. Similar conclusions can be drawn for the real backgrounds. It should be noted that while MEME and Weeder perform poorly in one of the two backgrounds, GALF-G maintains the competitive performance well in both.

**Table 7:** Experimental results on the improved eukaryotic benchmark.

**Algorithms**	**Markov**				**Real**			
		
	**nSn**	**nPPV**	**nPC**	**nCC**	**nSn**	**nPPV**	**nPC**	**nCC**
GALF-G	0.117	0.184	0.102	0.138	0.116	0.156	0.095	0.126
MEME	0.115	0.107	0.077	0.097	0.103	0.092	0.063	0.083
Weeder	0.133	0.043	0.032	0.052	0.202	0.071	0.055	0.096

Average performances (*nSn*, *nPPV*, *nPC *and *nCC*) of GALF-G, MEME and Weeder on the algorithm benchmark suite (50 datasets with Markov backgrounds and 50 with real backgrounds).

In the improved eukaryotic benchmark [35], which is considered more suitable to test motif discovery algorithms, GALF-G shows superior performance to the widely-used MEME and Weeder, while only top-scored motifs are compared. However, as stated in [33], it is more meaningful in practice to provide multiple motifs for testing [5] where the experiments are reported as following.

### Multiple Motifs Outputs on the *E. coli *Benchmark

In this sub-section, GALF-G was tested, to address a more realistic scenario, where multiple candidate motifs are desired for identifying the true TFBSs in biological research, on the *E. coli *benchmark. The *E. coli *benchmark ECRDB62A [34] has 62 datasets, on average about 300 bp in the sequence length varying from 86 to 676 bp, 12 sequences per dataset, around 1.85 sites per sequence and the average site width is 22.83 with standard deviation > 10, which indicates very diversified widths.

Specifically, minimal parameter-tuning policy was employed as if the programs were run by a common user with minimum prior knowledge in practice. Results of AlignACE [20], BioProspector [19], MDScan [5], MEME [12], MotifSampler [21] and Weeder [16] were obtained for comparison. A uniform width of 15 was input for those fixed-width algorithms, namely AlignACE, BioProspector, MDScan and MotifSampler. On the other hand, MEME was run with the default setting for widths and the optimal one was chosen automatically within. Weeder was run with the large width mode. For GALF-G, we ran it on the benchmark datasets with both the uniform fixed width 15 and also the widest range accepted for the program of *R *= [10,20] with |*R*| = 10 around the central width 15. For all algorithms, 5 motifs were output for detailed comparisons.

We employ the evaluation criteria from [34], namely precision *PPV*, sensitivity *Sn*, performance coefficient *PC *and *F*-score *F*, on both nucleotide (prefix *n*) and site (prefix *s*) levels [see Additional file 1] (We use the standard notation of PPV instead of the non-standard specificity definition in their work). In the comparisons shown in Table 8, the accuracy of the best prediction out of the top 5 scoring predictions is evaluated with respect to *nPC*. With both fixed-width and range inputs, GALF-G outperforms the other algorithms in all evaluation criteria. For example, GALF-G (15) outperforms the best among the other algorithms by 49% in *nPC*, 29% in *nF*, 28% in *sPC *and 18% in *sF*. GALF-G (rg), with width range input [10,20], outperforms the other best algorithms by 46% in *nPC*, 29% in nF, 25% in *sPC *and 24% in *sF*. By comparing the two different input settings for GALF-G we can see that with little sacrifice in other measures (< 0.01 on the nucleotide level and < 0.02 on the site level), the generalized model based on the range (rg) demonstrates improved site level sensitivity, in particular 15% (or 0.082) in *sSn *compared with GALF-G (15) and 34% (or 0.172) compared with the best among other algorithms.

**Table 8:** Experimental results on the *E. coli *benchmark.

**Algorithms**	**Nucleotide level (n)**				**Binding site level (s)**			
	**nPC**	**nSn**	**nPPV**	**nF**	**sPC**	**sSn**	**sPPV**	**sF**
GALF-G (15)	**0.260**	0.290	**0.309**	0.300	**0.386**	0.538	**0.520**	0.529
GALF-G (rg)	0.254	**0.297**	0.304	**0.301**	0.379	**0.620**	0.502	**0.555**
AlignACE	0.128	0.198	0.152	0.172	0.234	0.355	0.335	0.345
BioProspector	0.174	0.205	0.270	0.233	0.294	0.424	0.374	0.397
MDScan	0.149	0.177	0.230	0.200	0.240	0.328	0.355	0.341
MEME	0.158	0.259	0.199	0.225	0.295	0.461	0.436	0.448
MotifSampler	0.153	0.179	0.237	0.204	0.302	0.331	0.476	0.390
Weeder	0.152	0.162	0.204	0.181	0.307	0.543	0.387	0.452

Prediction accuracy on the ECRDB62A benchmark of *E. Coli *at nucleotide, binding site levels. GALF-G (15) was run with the fixed width 15 and GALF-G (rg) was run with the range [10,20]. The best results are bold.

Besides the best predictions out of the 5 outputs, investigation was also done to analyze the top-scored motifs as well as the rest individually for different algorithms. The statistics in terms of *nPC*, which reflects both *nPPV *and *nSn*, are shown in Table 9. As indicated before in [34], the top-scored predictions are not necessarily the best predictions, implying that outputting only a single prediction may not be a good choice in practice or for comparison studies. However, the top-scored predictions from GALF-G are significantly better than the best among the other algorithms, by 30% (w15) and 36% (rg) respectively. We can also see that, for GALF-G, the generalized model based on the range provides better performance than on the fixed width, with respect to both the top-scored and the mean predictions. This implies that the generalized model using ranges is useful when the prior width information is usually not strong in practice. On this benchmark for multiple motif outputs, GALF-G outperforms other state-of-the-art algorithms considerably. The generalized model exhibits improved sensitivity while maintaining competitive precision, and thus achieves better overall performance on the site level.

**Table 9:** Statistics on the *E. Coli *benchmark.

**Algorithms**	**Best**	**Worst**	**Mean**	**STD**	**Top-scored**
GALF-G (15)	0.260	0.094	0.121	0.031	0.169
GALF-G (rg)	0.254	0.080	**0.129**	0.040	**0.177**
AlignACE	0.128	0.029	0.072	0.045	0.083
BioProspector	0.174	0.097	0.124	0.041	0.130
MDScan	0.149	0.068	0.106	0.034	0.099
MEME	0.158	0.002	0.054	0.069	0.116
MotifSampler	0.153	0.010	0.062	0.065	0.069
Weeder	0.152	0.031	0.081	0.106	0.064

The statistics of the top 5 predictions in terms of *nPC *on the ECRDB62A benchmark. GALF-G (15) is run with the fixed width 15 and GALF-G (rg) is run with the range [10,20]. STD is the standard deviation. The best mean and top-scored results are bold.

### Multiple Motif Types in Real Datasets

In gene regulation, TFBSs of different kinds of motifs may appear in the same promoter region. They either work together to regulate the transcription or compete for the TF binding when part of the TFBSs overlap with each other. Thus it is meaningful to discovery multiple TFBS motifs, possibly with overlaps in some of their TFBSs, from a dataset simultaneously. The following experiments tested GALF-G under the corresponding scenario.

#### The liver-specific dataset

The liver-specific dataset [47] contains 19 sequences, embedded with several major motifs (with 6-19 sites) varying in widths, namely HNF-1, HNF-3, HNF-4 and C/EBP, and some other motifs with fewer sites, such as CRE, BRF-3 and BRF-4 with only one occurrence for each of them. Some TFBSs from different types of motifs overlap with each other in the dataset. For example, a TFBS of HNF-1 (width 15) overlaps with a TFBS of HNF-4 (width 12) with 7 bp in a particular sequence, while co-occurring TFBSs of HNF-1 and HNF-4 in some other sequences do not overlap at all. The total number of (overlapping) TFBS instances is 60. The widths vary dramatically from 7 bp to 31 bp.

On this dataset, GALF-G, GAME and MEME were compared using the width range input *R *= [8,16], which is considered a common range for TFBSs, to discover different types of motifs. The expected width for GAME was 12, the mean of the input range. Different numbers of motifs, *K*, ranging from 5 to 20 with step 5, were output and evaluated.

**Figure 2:**
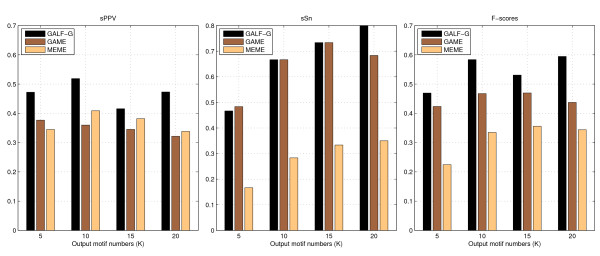
**Results on the liver dataset**. The results of precision (*sPPV*), recall (*sSn*) and *F*-scores (*sF*) with shift restrictions for different number of output motifs (*K *= 5, 10, 15, 20) on the liver-specific dataset.

The site level (with shift restrictions) results of *sPPV*, *sSn *and *F*-scores *sF *(with shift restrictions) based on all TFBSs are shown in Figure 2 for different *K*. MEME fails to produce comparable recalls or *F*-scores to the others. It is probably caused by the masking techniques not allowing overlapping of motifs. GAME masks TFBSs individually rather than the whole motifs, so better *sSn *(recall) can be obtained from a diverse GA population. With overlapping control on the GA, GALF-G shows recalls comparable to or better than GAME. Moreover, GALF-G has the best *sPPV *(precision) while GAME generally has the worst. Both GALF-G and MEME show an increasing trend of recalls as *K *increases. The sudden drop of GAME for *K *= 20 is probably because the expected width no longer suits some of the motifs while GAME actually performs fixed-width search in its GA. GALF-G provides the best balance between precisions and sensitivities, and thus gives the best *F*-scores in all cases. Averaged on all *K*, the *F*-scores are: GALF-G: 0.54, GAME: 0.45 and MEME: 0.31 where GALF-G outperforms the other two by 20% and 73% respectively.

Besides the previous evaluation that treats all the TFBSs as a whole, type specific investigation was also carried out on the output results of GALF-G. With the help of STAMP [48], the predicted motifs with *K *= 5 GALF-G were searched for matches of annotated TFBS motifs from the TRANSFAC database V11.3, based on ALLR (Average Log Likelihood Ratio). ALLR was considered to be the most effective in comparisons of single columns for motifs [48].

**Figure 3:**
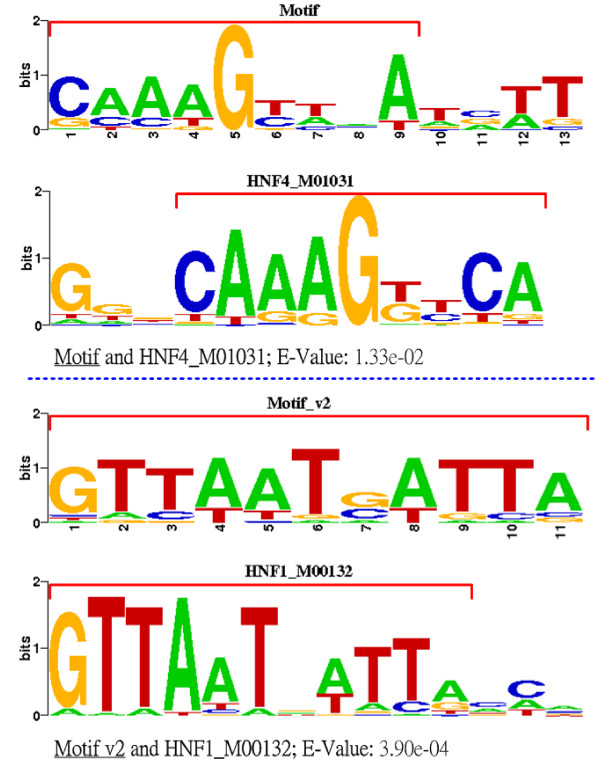
**TFBS matches of the liver dataset**. The matches from TRANSFAC for the top 2 high-scored motifs. The red brackets indicate the aligned blocks.

The relevant matches for the top 2 motifs are displayed in Sequence Logo formats in Figure 3. The top 2 high-scored motifs, labeled in STAMP by Motif (width: 13) and Motif v2 (width: 11), match HNF-1 and HNF-4 in TRANSFAC respectively with high statistical significance, i.e., low E-values (< 0.05). For Motif v4 (width: 16), it matches part of HNF-3 alpha without high statistical significance (E-value 2.71e-01), because only part of the HNF-3 TFBSs are identified in the predicted motif. It indicates that, top-scored motifs output by GALF-G in general match true TFBS motifs with high confidence. The other two motifs do not have relevant top 10 matches in TRANSFAC. C/EBP cannot be discovered as a whole motif, possibly due to its low conservation compared to the HNF motifs. STAMP also provides the phylogenetic profile where Motif (HNF-1) and Motif v2 (HNF-4) are grouped together, and so is Motif v4 (HNF-3), implying they belong to the same HNF family. For *K *= 10, similar results are obtained, with matches mainly including HNF-1 and HNF-4.

#### In-depth investigation on the MyoD dataset

The MyoD dataset seems to be an exceptional case among the 8 real datasets tested by GAME [27]. Only GALF-G (*sPPV*: 19/22, *sSn*: 19/21, *sF*: 0.88) and GALF-P (*sPPV*: 21/37, *sSn*: 21/21, *sF*: 0.72) are able to show acceptable site level results (with shift restrictions) in the fixed-width (*w *= 6) experiments, while in the variable width experiments none of the programs succeed in providing good results.

**Figure 4:**
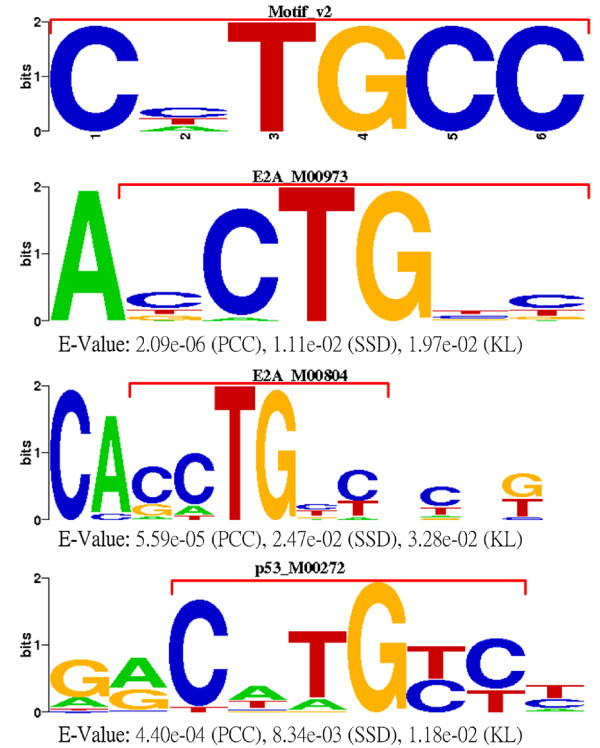
**TFBS matches of the MyoD dataset**. The matches from TRANSFAC to the 2nd motif output by GALF-G on the MyoD dataset. The red brackets indicate the aligned blocks.

To investigate into this exception, GALF-G was set to output *K *= 3 different motifs with the annotated width 6. Besides the fittest output being the annotated MyoD motif, the other two are only marginally lower in their fitness compared to the best one (differences < 2%). That is probably the reason why most existing algorithms perform poorly in this dataset - they either locate a sub-optimal because of the low signal-to-noise ratio, or obtain inappropriate rankings of the motifs due to the subtle differences in the modelling. It indicates that the accurate width information is still crucial for such subtle and short motifs. We searched the 2nd ranked motif, Motif v2, for matches from the TRANSFAC Database using STAMP, based on the various column comparison metrics provided by STAMP. Consistent matches, such as E2A [49,50], p53 [51,52], E47 [53] and E-box [54] motifs, were obtained with high rankings (within top 10s), and these motifs are closely related to MyoD for muscle cell regulation according to the references [49-54]. The most consistent matches are shown in Figure 4. Thus there is a high probability that Motif v2 is a true motif which may not have been annotated previously in the MyoD dataset. In summary, GALF-G outperforms GAME and MEME by 14% and 73% on average in *sF *respectively on the liver-specific dataset for multiple motifs discovery. Additionally, GALF-G sheds light to an additional motif which may not have been annotated previously in the MyoD dataset.

### Efficiency Experiments

**Figure 5:**
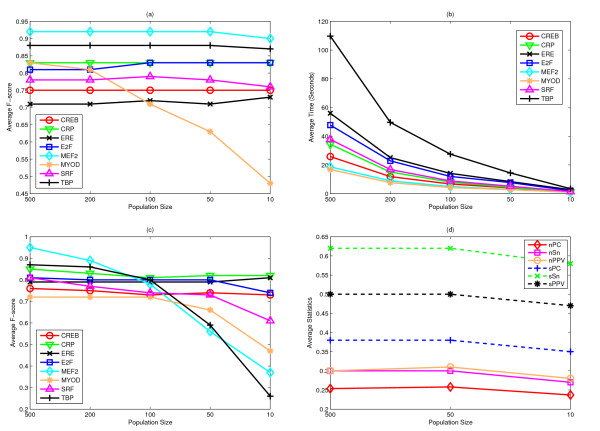
**Results with different population sizes**. Different population sizes: (a) The average site level *F*-scores *sF *of GALF-G on the 8 real datasets with fixed width inputs. (b) The average time of GALF-G according to (a). (c) The average *F*-scores of GALF-P on the 8 real datasets with fixed width inputs. (d) The statistics on both nucleotide and site levels on ECRDB62A of GALF-G with range inputs.

Although the effectiveness is the major concern for motif discovery, practitioners also prefer efficient algorithms which have capability for large scale data. In this sub-section, we tested GALF-G with different GA population sizes to investigate the trade-off between effectiveness and efficiency of meta-convergence. Firstly, different population sizes (*PS *= 500 (default: In the previous work, in order to be consistent with GAME's *PS *= 500, GALF-P employed the same setting as default, and this is followed in GALF-G for the minimum parameter-tuning purpose), 200, 100, 50, 10) were used to run GALF-G, GALF-P and GAME (results from [28]) on the 8 real datasets [27] for fixed-width single motif discovery. For each *PS*, they were run 20 times on the same Pentium D 3.00 GHz machine with 1 GB memory, running Windows XP, and the results were averaged. The effectiveness (site *F*-scores *sF*) and efficiency are shown in Figures 5 (a) to 5 (c). For the default *PS *= 500, the average time (in seconds) follows that: GALF-G (43.38) < GALF-P (61.91) < GAME (291.11). Since the standard deviation of GAME's effectiveness is already large with *PS *= 500, we only focus on GALF-G and GALF-P to compare the effects (except the special MyoD case better to run with *K *> 1) of different *PS*. In Figure 5 (a), the overall performance for *PS *= 500 are similar, as well as the standard deviations: GALF-G 0.004; GALF-P 0.029. However, when the population size drops to *PS *= 10, the performance of GALF-P drops significantly, and the standard deviation becomes 0.17 on average, and even ≥ 0.40 for MEF2 and TBP datasets (Figure 5 (c)). On the contrary, the average performance of GALF-G is maintained, and the overall standard deviation is only 0.031, still a very small number. Furthermore, the average time of GALF-G for *PS *= 10 is just 1.80 seconds, which is over 24 times speedup of the default *PS*, as shown in Figure 5 (b).

It is interesting that even with a population size of 10, GALF-G still performs comparably well, while GALF-P degenerates significantly. The major reason is due to the meta-convergence framework with similarity test, which is not used in GALF-P. With an extremely small population, GALF may not be able to provide the optimal motif in every run. However, since different motifs are controlled and maintained on a meta level in GALF-G, converged sub-optimal motifs will be replaced by better ones and eventually the global optimum can be found.

The above results imply that, GALF-G is able to provide comparable and consistent performance for fixed-width single motif discovery with a small population for competitive efficiency.

On the *E. coli *benchmark for multiple outputs (*K *= 5) with range inputs, we observed similar performance maintenance with different *PS *for GALF-G in Figure 5 (d), thanks to the meta-convergence mechanism to maintain different optimal motifs in the solutions. The average time on each dataset for the three *PS *is 655.80 (500), 74.40 (50) and 16.05 (10) seconds respectively, where the *PS *= 10 demonstrates a speedup of over 40 times compared to that of the default size (*PS *= 500). For *PS *= 10, the standard deviation of *nPC *is 0.0098, which is still small compared with 0.0070 for the default *PS*.

According to the efficiency experiments, GALF-G is able to maintain competitive effectiveness with very high efficiency. Therefore GALF-G has great potential to work on ever larger scale datasets successfully.

## Discussion and Conclusion

To conclude, we summarize the proposed work of GALF-G, discuss about the challenges and point out future directions.

### Summary

In this paper, the generalized motif model is proposed for realistic motif discovery problems. It models a possible range of widths rather than any single width. The model has the potential to address the biological uncertainty better and is more practical in reality because TFBSs of the same motif may vary in widths and exhibit different degrees of conservation. The meta-convergence framework is proposed to support multiple and possibly overlapping optimal motifs, based on the flexible and easy control of the similarity test for users. GALF-G is developed by incorporating the extended GALF searching methodology into the meta-convergence framework based on the generalized model.

GALF-G has been tested extensively on over 970 datasets, including 800 synthetic datasets, 8 real datasets (further 24 range cases), 100 eukaryotic and 62 *E. coli *benchmark datasets, as well as a real liver-specific dataset with multiple overlapping motifs. GALF-G has shown its competitiveness and better effectiveness for different kinds of motif discovery problems with both fixed-width and range inputs. The generalized model not only predicts the motifs accurately but also include more correct TFBSs. The searching capacity for optimal solutions and efficiency of the meta-convergence framework have also been demonstrated with the synthetic and real datasets. GALF-G has also discovered an additional motif which might not have been annotated previously in the MyoD dataset.

## Discussion

However, the motif discovery problem remains challenging due to the weak underlying motif signals input data, as well as the diversity and complexity of TF binding TFBSs [55]. There are also a number of potential improvements for the generalized motif model and GALF-G in our future work, such as further analysis on the effect of different width ranges, more efficient evaluation when handling different width fragments, flexible width distributions for different motif types, validation of the putative motif in MyoD dataset, etc. The candidate fixed-width model for the generalized model still needs more investigation to better suit the biological observation. Integrating the generalized model for motif discovery with additional evidence such as expression data to increase the prediction power is another attractive research direction to us.

## Methods

### The Proposed Model and Evaluation

#### Denotations and Formulations

With our focus on the matrix representation (PFM), the motif discovery problem is formulated as follows. Defined on the alphabet Σ = {*A*, *T*, *G*, *C*} for DNA sequences, the input data are a set of sequences *S *= {*S*_*i*_|*i *= 1, 2, ..., *m*}, where each *S*_*i *_is a sequence with length *l*_*i *_of nucleotides from the alphabet. The motif width *w *is assumed to be known for the time being. TFBS instances are represented by *R *= {} where each  is the *k*th instance of width *w *in *S*_*i*_. If we assume each sequence has at most one instance (ZOOPS), then  is collapsed to be *r*_*i *_(*r*_*i *_= null if *k *= 0) for short. Table 1 illustrates an artificial example of motif discovery. A site indicator matrix (SIM) *A*, which is also used to represent the solution, locates the TFBS instances as sites, where *A*_*ij *_= 1 if a motif instance (site) starts at position *j *of *S*_*i *_and 0 otherwise. Alternatively, we can use the position  = *j *to represent a instance  given *w*. Thus we have a compact position representation of *A *= {*p*_1_, *p*_2_, ..., *p*_*m*_} especially for ZOOPS, where some the positions can be NULL. A profile of the motif can be built from aligning the TFBS instances indexed by *A*. The profile is represented as a 4 × *w *Position Frequency Matrix (PFM) Θ, where Θ_*jb *_is the frequency of nucleotide *b *in column *j *of the motif. The nucleotides from background (non-motif sites) are represented by Θ_0_, where Θ_0*b *_is the frequency of nucleotide *b *in the background and is treated as known from the input.

The motif discovery problem (of a known width *w*) can be thus formulated as finding *A *(with only the TFBS sites being considered) and the corresponding PFM Θ such that one of the above scoring/fitness functions is maximized according to different assumptions.

#### The Probabilistic Models

To complete our generalized model, the important component comes from the existing models handling a known width input. In this paper, we employ the probabilistic models which have most intuitive explanation with the generalized model. For a candidate solution *A *(which also indicates Θ), the full Bayesian model of likelihood [13,40] can be written as

(5)

where Θ is the motif PFM, Θ_0*b *_is the background distribution of nucleotide *b*, *n*_*jb *_is the count of nucleotide *b *in column *j *of the PFM, *n*_0*b *_is the count of nucleotide *b *in the background, |*A*| is the total number of sites in the motif,  is approximately the number of all possible sites (the number of invalid sites is trivial and can be ignored), and *p*_0 _= |A|/L* is the estimated abundance ratio which represents the probability of any position being a site in the dataset. Θ_*jb *_= *n*_*jb*_/|*A*| (strictly it should be  as an estimate, but we just use Θ_*jb *_for simplicity). Similarly Θ_0*b *_≈ *n*_0*b*_/L* (ignoring the relatively small affect of *A*).

In Bayesian analysis, noninformative priors of the independent *p*(Θ) and *p*(*p*) are integrated out for convenience. Alternatively, by assuming them as constant we have the log likelihood as follows:

(6)

By ignoring the constant parts and approximating L* log(1 - *p*_0_) ≈ - L* * *p*_0 _= - |*A*| since *p*_0 _is very small, the equivalent score *psi' *can be written as

(7)

which is exactly the approximation form used in the Bayesian analysis [40]. With one step further to ignore the penalty of - |*A*|, we have the approximation form for a known *p *[40] and it is also coined as the Kullback-Leibler divergence with parameter (we use this form in the generalized model since we find the previous one imposes too much penalty on the number of TFBSs):

(8)

Furthermore, if we assume each sequence *S*_*i *_has exactly one site, i.e. one occurrence per sequence (OOPS), then *p*_0 _also becomes constant. As a result we only have to consider part of Equation 8

(9)

which is the well known information content (*IC*) [11]. *IC*(*j*) is defined as the positional *IC *for column *j*.

#### The Fitness Function and Evaluation

Recalling the generalized model in Equation 1, we can now choose *P*(*A*(*w*_*i*_) *|w*_*i*_) = exp(*ψ*(*w*_*i*_)) accordingly from the previous probabilistic models, where *ψ*(*w*_*i*_) is a simplified notation for exactly *ψ *(Θ, *A*|*S*, Θ_0_) in Equation 8 given *w*_*i*_. For computational convenience, we represent the fitness function *f *in log likelihood form as

(10)

In the evaluation, a candidate solution consists of *A *(and the derived Θ) with the maximal width *w*_*max*_. For each particular *w*_*i *_from the range *R*, we have to choose the fragment (a continuous *w*_*i*_-submatrix *A*(*w*_*i*_) from the full matrix Θ) that maximizes *ψ*(*w*_*i*_) (see Figure 1). It is equivalent to maximizing *IC *for width *w*_*i *_since *p *in Equation 8 is now fixed for all *A*(*w*_*i*_). With the log format of *f*, we can avoid overflow with the *exp *function by taking out the largest log component during mediate computation and adding it back upon finishing the evaluation.

For the convenience of implementations of searching and consistency with other methods for evaluation (which output single-width motifs), a core fragment, located by the width *w*_*cor *_and offset *w*_0_, is to be selected. *w*_*cor *_and *w*_0 _are also determined based on *IC*. Starting from the two ends of the maximal PFM with *w*_*max*_, we iteratively remove each columns *j *with positional *IC*(*j*) lower than the average. The remaining submatrix (or *A*(*w*_*cor*_)) is thus with width *w*_*cor *_and offset *w*_0_. Complexity of the whole evaluation grows quadratic to |*R*| = *w*_*max *_- *w*_*min *_+ 1. Since the ranges are usually restricted within 5 - 10 bp, *f *is computationally feasible in practice with additional *O*(|*R*|^2^) overhead compared with a fixed width model for *w*_*max*_. The offset *w*_0_, combined with the position *p*_*i *_of *A *in the *i*^*th *^sequence, is also used to determine the aligned position ( (*A*)) in the similarity test in Equation 2.

### Meta-convergence Framework Details

#### Similarity test settings

The shift tolerance in Equation 2 is set as *tol *= 3 + (|*R*| - 1)/2. The first part of *tol *is chosen for convenience to separate two TFBSs and the latter part is the tolerance for the range involved. In the case of competition for the same slot in slot dispatching, the threshold can be flexibly specified by the users (for general usage, the default is: *st *= 0.3, which is used throughout this paper). Users can customize *st *based on their needs, either with a large value (e.g. ≥ 0.5) to force solutions of highly different motifs, or with a small value (e.g. ≤ 0.1) to allow fine variations of the same motif type. On the other hand, for deleting individuals in the case of near convergence, the threshold is automatically fixed at the value of *st' *= 0.5 to make room for the other solutions. *st' *is not sensitive because the similar optimal motifs are finally controlled by the user-specified threshold *st*. However, if *st' *is set to be too low, many similar variations to the converged motif will remain in the population, and time will be wasted to converge repeatedly to the same motif kind.

#### Meta-convergence

**Figure 6:**
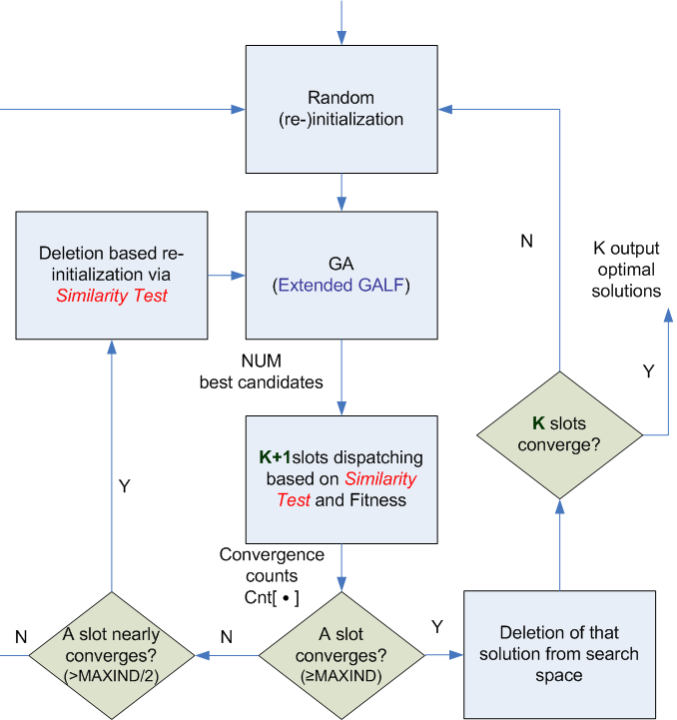
**The procedure of meta-convergence**.

In greater detail, the meta-convergence framework can incorporate any GA procedure (Genetic Algorithm with Local Filtering (GALF) [28] in our case). Like in the previous approaches [27,28], up to a maximum number of the GA executions, MAXRUN, can be run but it will stop running if the convergence test is satisfied. Additionally in meta-convergence, K+1 slots are maintained where K is the number of optimal solutions expected. Each slot stores the best solution of a different of motif kind, and is allocated a counter Cnt, which keeps track of its motif convergence count. At the end of each GA run, a number (NUM) of best solutions (individuals) will be dispatched and subject to the similarity test to the K+1 slots. The corresponding counter will increment for each update of a solution of the same motif kind and reset if the motif is replaced by a new one. A convergence threshold MAXIND is used to monitor convergence. MAXIND is a relatively small number because each dispatched solution is already a converged one obtained by GA. In general, the meta-convergence framework needs at most MAXRUN GA runs to obtain K optimal solutions while the previous methods such as GAME and GALF-P need K*MAXRUN runs. The whole procedure of meta-convergence is illustrated in Figure 6.

#### Similarity test applied in the framework

Solutions that pass the similarity test, i.e. those belong to the same motif kind in a particular slot, will compete for the same slot based on their fitness. On the other hand, the solution of a new motif will occupy an empty slot or the slot storing the solution with the worst fitness. After each GA run, when a slot is near convergence (we define this situation as Cnt > MAXIND/2), solutions similar to it will be eliminated, again based on the similarity test, to make room for the other optimal solutions in the next GA run. When the solution of a particular motif in the slot has converged (i.e. Cnt ≥ MAXIND), the motif will be taken out from the search process, i.e. all the exactly matched TFBSs belonging to this motif will be deleted, making room for efficient discovery of other motifs. The extra (K+1)^*th *^slot is used to keep certain sub-optimal solution in the early stage in order not to lose them, because otherwise the Cnt may fluctuate especially for the K = 1 case when there are several motifs with close fitness competing for the only slot.

### GALF-G Implementations

We employ the genetic algorithm (GA [see Additional file 1]) based GALF [28] as the searching procedure. However, since GALF was previously based on simpler assumptions, it has to be extended accordingly to suit the need of the generalized model.

#### Extended GALF Operators

Local filtering (LF) is the feature operator of GALF, which employs the combined representations for the whole motif (PFM Θ) and individual instances (SIM *A*). However, it was based on the simple OOPS and fixed-width assumptions. As a result, extensions have to be made for more general cases addressed by GALF-G.

Generally, LF refines each individual (candidate solution) by iteratively scanning the sequence containing the currently worst instance and choosing the best replacement. To evaluate each instance (site) of the individual, the similarity score with the consensus concept is proposed. However, the relation between this heuristic score and the fitness is implicit. In GALF-G, we propose to use the log likelihood ratio for an instance fragment starting at the  column with width *w'*,

(11)

to evaluate each instance *r*_*i*_, where *r*_*i*_(*j*) ∈ Σ is the nucleotide in column *j *of *r*_*i*_,  is the corresponding frequency from the PFM and  is the corresponding background frequency. It measures the ratio of *r*_*i *_generated by the motif PFM over the background, and is more closely related to *ψ *(*w*_*i*_) in Equation 10. The effectiveness of the log likelihood ratio and the mutation operator are verified [see Additional file 1] on the 8 datasets tested in [27]. In range input cases, with the *w*_*cor *_core fragment stored, we encourage LF to match instances with a longer width (≥ *w*_*cor*_) so that the width *w' *is chosen randomly from [*w*_*cor*_, *w*_*max*_] and thus LF can be applied with fewest modifications.

Because now the fitness *f *can handle the general case with any motif instances, the new GALF-G can now search based on zero or one occurrence per sequence (ZOOPS) assumption rather than OOPS. However, it is unwise to randomly generate null positions for non-sites at the very beginning during searching. It is because when most of the individuals are poor in their fitness, fewer instances will be strongly biased and the population will suffer from undesirable premature convergence. To alleviate this problem, we initialize the population with OOPS assumption and refine the abundance ratio (*p*_0 _in Equation 8) in later generations using a new mode of LF. The convergence (CONVER) mode of LF is triggered when the best individual stagnates for more than 1/4 of the convergence count MAXCONVER, or when it is toward the maximal generation of the GA. The convergence mode LF is applied to all individuals to adjust the motif abundance. The procedure is similar to normal LF except that the full *w*_*max *_fragment will be chosen for each instance and the worst instances are to be removed rather than refined, if eliminating it makes the overall fitness *f *increase.

#### Other Extensions

We adopt the single-point mutation and pre-selection from GALF-P [28] and choose multi-point (close to uniform) crossover instead of single-point because it provides higher diversity. Since the new model adjusts widths automatically, the shift operator in [28] is no longer needed.

To handle general cases other than the ZOOPS assumption, where there may be several occurrences in a sequence, we employ a refinement process for additional instances upon the meta-convergence of GALF runs. Generally, if a fixed width is input, instances have to increase *f *in order to be added, while in the width range case, the threshold of *f *is relaxed slightly [see Additional file 1 for the details].

Combining the meta-convergence framework with extended GALF based on the generalized model, as well as the refinement procedure, we have the proposed GALF-G to discover multiple TFBS motifs [see Additional file 1 for the pseudo-codes of the new LF, the extended GALF and GALF-G].

### Parameter Setting

Besides the parameters discussed specifically (such as motif widths and output motif number *K*), and except the efficiency experiments (with different *PS*), the other parameter setting exactly follows GALF-P [28] with the purpose of minimum tuning. In the extended GALF: default population size *PS*: 500; maximal number of generations MAXGEN: 300; interval of generations to trigger local filtering (LF)-INTL: 10; convergence count MAXCONVER: 50; mutation rate: 0.9; crossover rate: 0.3; and maximal runs of GALF MAXRUN: 20. The quite large population size follows the setting of GAME for fair and consistent comparisons, though it turns out that a smaller population size also works comparably well (in the efficiency experiments).

## Authors' contributions

TMC proposed the ideas, developed the algorithm and interpreted the results. GL refined the ideas and implementations, and carried out the experiments for comparisons. KSL and KHL were involved in the design and supervision of the project. TMC, KSL and KHL jointly wrote the manuscript. All authors read and approved the final manuscript.

## Supplementary Material

Additional file 1 **Supplementary materials for discovering multiple realistic TFBS motifs based on a generalized model**. Supplementary materials of additional details about implementations, datasets and experiments. Click here for file
