# Supplementary material for: Discovering multiple realistic TFBS motifs based on a generalized model
Source: BMC Bioinformatics. 2009 Oct 7;10:321. doi: 10.1186/1471-2105-10-321 (PMC2770069; doi:10.1186/1471-2105-10-321)
Supplement: Additional file 1 — Supplementary materials for discovering multiple realistic TFBS motifs based on a generalized model. Supplementary materials of additional details about implementations, datasets and experiments. [file 1471-2105-10-321-S1.PDF]

Supplementary Materials for Discovering Multiple Realistic TFBS Motifs Based on a Generalized Model

Tak-Ming Chan*, Gang Li, Kwong-Sak Leung, Kin-Hong Lee

Department of Computer Science & Engineering,
The Chinese University of Hong Kong,
Shatin, N. T., Hong Kong

*To whom correspondence should be addressed. Email: tmchan@cse.cuhk.edu.hk

1 Evaluation Measurements

This section corresponds to the evaluation measurements mentioned in the section: **Experiments** of the paper.

1.1 Site Level Evaluation Measurements with Shift Restrictions

Before going into details of the experiments, we will first describe the evaluation measurements for the experiments except the eukaryotic [1, 2] and *E. coli* [3] benchmarks.

The measurements are based the site level with shift restrictions. In general, we use the similar evaluation measurements following GALF-P and GAME. The site PPV (positive prediction value or precision [1]) $sPPV$, and site sensitivity (recall) sSn are defined as follows:

$$sPPV = \frac{sTP}{sTP + sFP},\ sSn = \frac{sTP}{sTP + sFN} \tag{1}$$

where sTP (true positive) indicates the number of the correctly predicted sites, sFN (false negative) indicates the number of the true sites which are missed in the prediction, and sFP (false positive) indicates the number of the wrongly predicted sites.

In previous benchmarking works [1, 3] on measuring the site level accuracy, sTP is the number of predicted sites which overlaps with the true binding sites by at least 1bp; sFN is the number of true binding sites that have no overlaps with any predicted sites; and sFP is the number of predicted sites which have no overlaps with the true binding sites.

Note that with the shift restrictions, we have a more strict criterion on sTP, where a predicted site can only shift no more than $3 + (|R| - 1)/2$ bp from the true site, where $|R|$ is the width range size of the input. We also restrict the sTP to be at least 1 bp overlapping with the true site. In a fixed-width case, the shift is 3 bp, according to the evaluation in [4] and [5]. Otherwise, the predicted site will be counted as an sFP and the missed true site will be counted as an sFN, respectively. These measurements reflect both site level performance and some degree of the nucleotide level accuracy in a concise way, and thus are chosen to evaluate all methods for most of the comparisons in our experiments.

[1] We follow the notations of benchmark papers [1, 3] for consistency.

Even in a wide range case, it is still more strict than the measurements in the previous works. In fact by varying the shifts from 3-8 bp we find that only a small portion of the results are affected and the rankings of the methods being compared do not change in general.

In addition, combining both $sPPV$ and sSn, the site level F-score sF [6] is defined as their harmonic mean as follows:

$$sF = \frac{2 * sPPV * sSn}{sPPV + sSn}. \tag{2}$$

If $sPPV = sSn = 0$, sF is set to 0. A high F-score indicates both the $sPPV$ (precision) and the sSn (recall) are high.

1.2 t-test for Performance Differences

To test whether the performances between two algorithms are significantly different given the experimental results (on randomly generated datasets or independent runs), the two-sample t-test with unequal variances (also called Welch's t-test [7]) is employed. The null hypothesis is that the means of two normally distributed populations (performances of the algorithms) are equal.

The t statistic to test whether the means $\overline{X}_1$ and $\overline{X}_2$ from two samples (experimental results) are different can be calculated as follows:

$$t = \frac{\overline{X}_1 - \overline{X}_2}{s_{\overline{X}_1 - \overline{X}_2}} \tag{3}$$

where

$$s_{\overline{X}_1 - \overline{X}_2} = \sqrt{\frac{s_1^2}{n_1} + \frac{s_2^2}{n_2}} \tag{4}$$

and s_1 and s_2 are the unbiased estimators of the standard deviations of the two samples, and n_1 and n_2 are the sample sizes. The degree of freedom DF is calculated as

$$DF = \frac{(s_1^2/n_1 + s_2^2/n_2)^2}{(s_1^2/n_1)^2/(n_1 - 1) + (s_2^2/n_2)^2/(n_2 - 1)}. \tag{5}$$

The t statistic and DF are then used in a one-sided test (the alternative hypothesis is one algorithm is better than the other) to decide the p-value, or the significance level of rejecting the null hypothesis.

1.3 Evaluation Measurements for the Benchmarks

We follow the evaluation measurements for the eukaryotic benchmarks [1, 2] and the *E. coli* benchmark in the corresponding experiments, namely on the side level: $sPPV$[2], sSn, sF and sPC; on the nucleotide level: $nPPV$, nSn, nF

[2] The authors of [3] used a non-standard notation of specificity, and we adopt the standard one as PPV for consistency.

(specifically in the *E. coli* one [3]), nPC and nCC (in eukaryotic ones [1, 2]), all of which will be explained below:

There is slight difference between our site level measurements with shift restrictions and those for the benchmarks. On the site level evaluation according to the benchmarks, the criterion for an sTP (true positive) is relaxed where at least 1bp has to overlap with the true binding site. Besides $sPPV$, sSn and sF defined in the previous sub-section, another measure of performance coefficient (PC) is employed, which shows similar property and the same ranking as the F-score [3]. On the site level, sPC is defined as:

$$sPC = \frac{sTP}{sTP + sFP + sFN} \tag{6}$$

Since the site level evaluation measures are relatively loose, additional measures are employed in the benchmark to depict the nucleotide level performance more accurately. For each site pair, i.e. a predicted site overlapping with a true binding site, the specificity (precision or PPV) $nPPV$ and sensitivity (recall) nSn on the nucleotide level are defined as

$$nPPV = \frac{nTP}{nTP + nFP}, \; nSn = \frac{nTP}{nTP + nFN} \tag{7}$$

nPC is defined in a similar way to sPC for a site pair:

$$nPC = \frac{nTP}{nTP + nFP + nFN} \tag{8}$$

For the eukaryotic benchmarks [1, 2], the correlation coefficient on the nucleotide level nCC, and $sASP$ average site performance, are defined as follows:

$$nCC = \frac{nTP * nTN - nFP * nFN}{\sqrt{(nTP + nFN)(nTN + nFP)(nTP + nFP)(nTN + nFN)}} \tag{9}$$

$$sASP = (sSN + sPPV)/2 \tag{10}$$

The average/overall measurements are slightly different between the eukaryotic benchmarks [1, 2] and the *E. coli* benchmark [3]. For the eukaryotic benchmarks, all overall measurements are calculated based on the total counts of TP, TN, FP and FN with the same formulas shown above, which are quite straightforward. However, for the *E. coli* benchmark, the averaging methods are based on all overlapping predicted-and-true site pairs, and are briefly described below:

For those predicted site which do not overlap with any true binding sites (missing predictions MP), and those true binding sites which do not overlap with any predicted sites (missing targets MT), they choose the larger one and add them into the denominator of site pairs. To get the average measures, the sum of nPC, $nPPV$ and nSn of site pairs are first averaged on the number of

site pairs ($\sharp SP$), and then on the number of sequences ($\sharp SEQ$) in each dataset, and finally on the number of datasets ($\sharp DS$), as shown below:

$$\frac{1}{(\sharp DS)} \sum_{\sharp DS} \frac{1}{(\sharp SEQ)} \sum_{\sharp SEQ} \frac{1}{(\sharp SP)} \sum_{\sharp SP} nPC \text{ (or } nPPV \text{ or } nSn) \tag{11}$$

Also, the F-score on nucleotide level is computed accordingly for the *E. coli* benchmark as:

$$nF = \frac{2 * nPPV * nSn}{nPPV + nSn} \tag{12}$$

Details of these measurements can be found in [3].

2 Single Fixed-width Motif Discovery on Synthetic Data

This section corresponds to the details in the sub-section: **Experiments: Single Fixed-width Motif Discovery on Synthetic Data** of the paper.

2.1 The Synthetic Data Generation

The synthetic data were generated with 8 scenarios from the following combinations of: (1) motif width: short (8 bp) or long (16 bp); (2) number of sequences: small (20) or large (60); and (3) motif conservation: high (dominant nucleotides generated with 0.91 probability) or low (in 60% columns dominant nucleotides were with 0.91 probability, while in 40% dominant nucleotides only with 0.55 probability). For each scenario, 100 random datasets with 300bp sequences were generated accordingly, embedded with the TFBS instances of a random motif. The number of TFBSs in a sequence followed a geometric distribution and a few sequences contain no instances. Details on generating the datasets were provided in [5].

2.2 The Complete Results of the Synthetic Experiments

Tables 1 to 4 show the complete results with $sPPV$ (or precision on the site level), sSn (or recall on the site level) and the F-scores sF of the 800 synthetic datasets experiments corresponding to Table 2 in the paper. BioOptimizer, based on MEME and BioProspector, makes little observable average difference from the two algorithms respectively.

2.3 t-test for GALF-G and MEME on the Synthetic Experimental Results

As expected, GALF-G and GALF-P are close in their performances. To investigate the performance differences between GALF-G and MEME, the latter of which seems to also show competitively close performances, the two-sample Welch's t-test [7] was employed. The respective p-values of GALF-G better than MEME, and MEME better than GALF-G, with respect to sF for the

Tab. 1: Average performance comparisons for the 800 fixed-width synthetic datasets experiments. For each scenario 100 datasets are generated. Width: the motif width, Num: the number of sequences and Con: conservation degree.

Scenarios	GALF-G			GALF-P		
Width /Num /Con	$sPPV$	sSn	sF	$sPPV$	sSn	sF
Short /Small /Low	0.50	0.51	**0.48** ±0.29	0.38	0.56	0.44 ±0.27
Short /Large /Low	0.58	0.51	**0.55** ±0.22	0.52	0.59	**0.55** ±0.22
Long /Small /Low	0.91	0.88	**0.89** ±0.13	0.87	0.91	**0.89** ±0.14
Long /Large /Low	0.94	0.89	**0.91** ±0.06	0.91	0.90	**0.91** ±0.05
Short /Small /High	0.83	0.84	0.84 ±0.07	0.73	0.90	0.80 ±0.09
Short /Large /High	0.84	0.81	**0.85** ±0.04	0.81	0.86	0.83 ±0.05
Long /Small /High	1.00	0.95	**0.98** ±0.02	0.97	0.99	**0.98** ±0.03
Long /Large /High	1.00	0.97	**0.99** ±0.01	0.97	0.97	0.97 ±0.02
Average	0.82	0.79	**0.81**	0.77	0.84	0.80

Tab. 2: Average performance comparisons for the 800 fixed-width synthetic datasets experiments (Continued)

Scenarios	GAME			MEME		
Width /Num /Con	$sPPV$	sSn	sF	$sPPV$	sSn	sF
Short /Small /Low	0.29	0.32	0.30 ± 0.30	0.49	0.34	0.39 ±0.35
Short /Large /Low	0.42	0.32	0.36 ± 0.30	0.63	0.33	0.42 ±0.29
Long /Small /Low	0.78	0.87	0.82 ± 0.22	0.91	0.86	0.88 ±0.14
Long /Large /Low	0.92	0.90	0.90 ± 0.07	0.96	0.85	0.90 ±0.07
Short /Small /High	0.71	0.80	0.75 ± 0.23	0.87	0.84	**0.85** ±0.07
Short /Large /High	0.83	0.83	0.83 ± 0.10	0.91	0.76	0.83 ±0.04
Long /Small /High	0.94	0.99	0.97 ± 0.03	0.98	0.99	**0.98** ±0.02
Long /Large /High	0.98	0.99	0.98 ± 0.01	0.99	0.98	0.98 ±0.01
Average	0.73	0.75	0.74	0.84	0.74	0.78

corresponding scenarios, are shown in Table 5. In 4 of the 6 scenarios where GALF-G shows better average sF (scenarios except 5, 7), GALF-G is better than MEME within the significance level 0.05. On the other hand, MEME shows no obvious significance of being better than GALF-G in the other 2 scenarios.

3 Single Motif Discovery on Real Datasets

3.1 Running Parameters for the Range Experiments

This section corresponds to the running parameter discussion in the sub-section: **Experiments: Single Motif Discovery on Real Datasets: (ii) (K = 1) variable-width (range) experiments** of the paper.

The input range is defined as $R_i = [w_{min((i)}, w_{max(i)}]$. Note that GAME in fact does not perform a variable width search in its GA, but only adjusts the width of the best individual in the post-processing. As a result, $\text{int}((w_{min((i)} + w_{max(i)})/2)$ was used as the expected width for GAME.

MEME searches repeatedly with the respective fixed width of each of the widths in the given range. After collecting all the candidates from each width, MEME evaluates the candidates of all possible widths using E-values [8]. As a result, the motif output in slightly different ranges may be identical, and the

Tab. 3: Average performance comparisons for the 800 fixed-width synthetic datasets experiments (Continued)

Scenarios	BioPros.			BioOpt.B.		
Width /Num /Con	*sPPV*	*sSn*	*sF*	*sPPV*	*sSn*	*sF*
Short /Small /Low	0.43	0.36	0.39 ± 0.31	0.44	0.36	0.39 ± 0.31
Short /Large /Low	0.58	0.37	0.45 ± 0.23	0.59	0.37	0.45 ± 0.23
Long /Small /Low	0.96	0.74	0.83 ± 0.14	0.96	0.74	0.83 ± 0.14
Long /Large /Low	0.98	0.68	0.80 ± 0.11	0.98	0.68	0.80 ± 0.11
Short /Small /High	0.82	0.75	0.78 ± 0.12	0.83	0.76	0.79 ± 0.11
Short /Large /High	0.88	0.68	0.76 ± 0.06	0.88	0.68	0.76 ± 0.05
Long /Small /High	1.00	0.94	0.97 ± 0.03	1.00	0.94	0.97 ± 0.03
Long /Large /High	1.00	0.92	0.96 ± 0.02	1.00	0.92	0.96 ± 0.02
Average	0.83	0.68	0.74	0.83	0.68	0.74

Tab. 4: Average performance comparisons for the 800 fixed-width synthetic datasets experiments (Continued)

Scenarios	BioOpt.M.		
Width /Num /Con	*sPPV*	*sSn*	*sF*
Short /Small /Low	0.49	0.34	0.39 ±0.35
Short /Large /Low	0.63	0.33	0.42 ±0.29
Long /Small /Low	0.91	0.86	0.88 ±0.14
Long /Large /Low	0.96	0.85	0.90 ±0.07
Short /Small /High	0.87	0.84	**0.85** ±0.07
Short /Large /High	0.91	0.76	0.83 ±0.04
Long /Small /High	0.98	0.99	**0.98** ±0.02
Long /Large /High	0.99	0.98	0.98 ±0.01
Average	0.84	0.74	0.78

range information, sometimes helpful to point out the conserved regions, may not be utilized at all. On the other hand, GALF-G attempts to search the more complicated search space where the motif width is considered collectively.

For FlexModule, the first-order Markov background was employed as suggested by the authors. We also tried both the default priors (better average $sF = 0.61$ achieved and shown) and the priors suggested by the author to strongly reduce reverse prior pseudo counts (average $sF = 0.55$, not shown). The width inputs were set according to the particular ranges R_i and $K = 1$ motif was output. Other parameters were set default.

Weeder is optimized with several range modes: small (widths 6, 8), medium (widths 6, 8, 10) and large (widths 6, 8, 10, 12). For each dataset, we chose the mode that is closest to the corresponding true witdh. As a result, small mode was applied on MyoD and TBP; medium mode was applied on CREB and MEF2; and large mode was applied on the rest datasets [3]

4 GALF-G Extensions for Extreme Cases

This section corresponds to the discussion on the Tompa et al Benchmark [1] in sub-section: **Experiments: Single Motif Discovery Challenges on Eukaryotic Benchmarks** of the paper.

[3] CRP width is 23 but the longest width mode that Weeder supports is large.

Tab. 5: The t-test p-values between GALF-G and MEME for the scenarios according to Tables 1 to 4. [ ] indicates the case when the counterpart is better in the average sF. Those p-values within the significance level 0.05 are shown in bold.

Scenarios	GALF-G better	MEME better
Short /Small /Low	**0.0246**	[0.9754]
Short /Large /Low	**0.0002**	[0.9998]
Long /Small /Low	0.3006	[0.6994]
Long /Large /Low	0.1397	[0.8603]
Short /Small /High	[0.8432]	0.1568
Short /Large /High	**0.0003**	[0.9997]
Long /Small /High	[0.5000]	0.5000
Long /Large /High	**0.0000**	[1.0000]

With an exploratory purpose for future works, we modified GALF-G with necessary extensions to handle those somehow exceptional violations in extreme cases, and present the preliminary results on the Tompa et al benchmark [1].

4.1 The Challenges Raised by Tompa et al Benchmark

The recent well-known eukaryotic benchmark by Tompa et al [1] deviated from the general premises of motif discovery and thus introduces extreme challenges. There are datasets, each of which is with fewer than 4 sequences and only one sequence, or even none, contains TFBSs. Many of the TFBSs, are poorly conserved (compared to some false "motifs" from the background) with respect to the current models. Motivated by this, improved benchmark has been proposed to categorize datasets that really have some motifs to test existing algorithms [2]. We also adopt the improved benchmark for testing in the main paper. In the Tompa et al benchmark, the long sequence lengths also complicate the problem by ever reducing the signal-to-noise ratios [3]. The motif discovery programs reported performed poorly ($nPC \leq 0.078$ [1]).

The probable reason is that some general premises have to be adopted for (sequence-based) motif discovery, e.g. GALF-G, to succeed. Firstly, there should be sufficient informative input data. The ZOOPS assumption would fail when there are only fewer than half of the sequences containing the target TFBSs. In fact, such input data should not be considered as reasonable nor acceptable, since the so-called underlying motif is even not representative for the data. Secondly, target TFBSs should be conserved to form certain motifs according to existing models. Significantly varying TFBSs are not likely to be identified, because the generalized model in GALF-G generalizes only current conservation-based models in this work. Nevertheless, in the future we seek novel models to describe TFBSs patterns better beyond conservation.

In the following sub-sections, we extend and test GALF-G on the Tompa et al benchmark as a preliminary attempt to handle such extreme cases.

4.2 Necessary Extensions

For extreme input data ($<$ 4 sequences), a more general assumption replaces ZOOPS, with other steps unchanged in principle; to handle poorly conserved TFBSs, GALF-G is run with multiple parameters and only those TFBSs being discovered most are output by voting. We will show below in detail the preliminary GALF-G extensions to handle (i) few informative sequences (data input) and (ii) poor conservation. The extensions are (i) horizontal Scanning and (ii) multiple-parameter voting, respectively.

4.2.1 Horizontal Scanning

For extreme input data with few sequences (< 4) and probably even fewer sequences containing target TFBSs (≤ 1), a more general assumption has to replace ZOOPS, to sample additional TFBSs in the few informative sequences.

Differently from OOPS or ZOOPS, in the initialization of each individual in the GA, a candidate instance (subsequence) i with w_{max} is selected from the input randomly, and then all input sequences are scanned to take into those instances within (loosely large) hamming distance $d = w_{max}/2$. Then these instances together form a motif individual (i.e. candidate solution). That is why we call the setting horizontal scanning. Accordingly, operators are kept consistent except being modified to accommodate the new representation. The slight difference is that we no longer need the refinement for additional instances due to the ZOOPS assumption in GA. This configuration is only applied in those extreme cases where the input sequence number < 4. The reason is that the computational cost will be unnecessarily high whereas we can take advantage ZOOPS for most common cases with enough informative input data.

To increase the signal strength in the Tompa et al benchmark with very long sequences ($>$ 2000bp) in contrast to the small sequence number, we follow the general procedure [4] to mask those repeat patterns with width ≥ 6 in advance.

4.2.2 Multiple-parameter Voting

To handle poorly conserved TFBSs, GALF-G is run with multiple parameters and only those TFBSs being discovered most are output.

The problem introduced by poorly conserved TFBSs is that the existing models usually rank false motifs from the background much higher than the degenerated ones formed from true TFBSs. Though this exhibits the demand for exploring unconventional models to describe motifs, some techniques may alleviate the problem, such as ensembles of several different algorithms and models[9, 10] and multiple-parameter voting (e.g. Weeder [11]). As a result, we adopt the latter one as a preliminary try because we focus on only the generalized model. In our setting, 5 motifs (with the default similarity threshold) are output with 7 different range parameters divided from $[6, 16]$, namely $[6, 8]$, $[8, 10]$, ..., $[14, 16]$. For each range, both OOPS and general (any number of occurrences per sequence) assumptions are set and tested. Each position in the input data being the TFBSs output in the results are voted. Finally we select

those TFBSs being discovered most as the outputs. In particular, we output those subsequences with $v_{threshold} \geq 1$ vote on average. Width constraints for such subsequences are also imposed, with minimal 5 and maximal 16.

We employ the aforementioned necessary extensions, and keep the framework unchanged in principle. Of course further investigations are needed if we want to achieve an optimal setting. Nevertheless, we choose the preliminary proposal to meet the exploratory purpose of testing GALF-G on the Tompa et al. Benchmark.

4.3 Preliminary Results

To investigate an appropriate setting, different vote thresholds $v_{threshold}$ were used from 0 to 100% of the maximal average vote v_{max} in each dataset, with a step size of 50%. The tradeoffs between $v_{threshold}$ and nSn, $nPPV$, nPC, sSn, $sPPV$ as well as $sASP$ are shown in Figure 1. As the threshold becomes higher, GALF-G in general will predict more accurately, while the sensitivity will decrease because fewer results will be output.

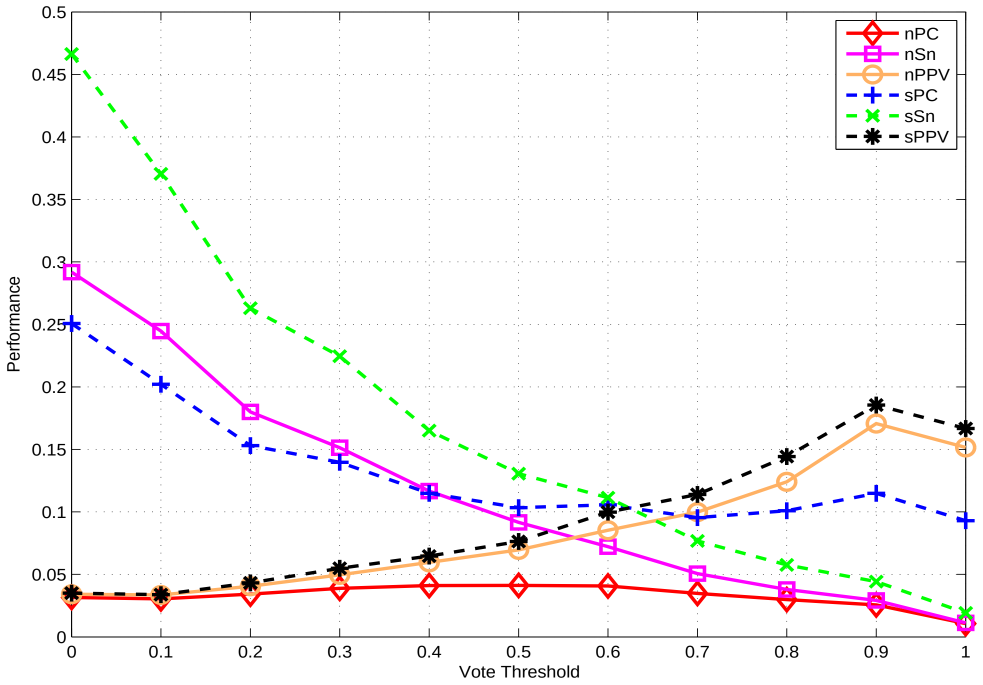

Fig. 1: The tradeoffs of nSn, $nPPV$, nPC, sSn, $sPPV$ as well as $sASP$, with respect to different $v_{threshold}$ from 0 to 100% of v_{max} on the Tompa et al benchmark.

Since many of the target motifs are poorly-conserved which cannot be correctly evaluated by the conservation based model we employ, the $v_{threshold}$ cannot be too larger in order to include positive TFBSs. As a result, large false positives are output and thus the precision (PPV) is low. There is no obvious trend of increasing PPV when we increase the $v_{threshold}$ for fewer outputs, indicating the target motifs cannot be appropriately measured by conservation. Finally, we select the middle $v_{threshold} = 50\%$ as an intuitive setting for the comparisons with other representative algorithms.

The comparisons in terms of nucleotide and site levels are shown in Table 6. GALF-G does not show very promising performance because there are many cases that GALF-G is not appropriate to handle, and the overall performance is based on the summed up TP, TN, FP and FN including these datasets. However, when we have a look at those datasets, of which the TFBS motifs are probably well conserved, that GALF-G can handle (with non-zero performances) in Table 7, we can find that GALF-G performs better than Weeder in more of such datasets, even with very stringent $v_{threshold} = 90\%$.

Tab. 6: Comparisons on the Tompa et al benchmark with representative algorithms [1] at nucleotide, binding site levels.

Algorithms	Nucleotide level (n)				Binding site level (s)		
	nSn	nPPV	nPC	nCC	sSn	sPPV	sASP
GALF-G	0.092	0.070	0.041	0.483	0.131	0.076	0.103
AlignACE	0.055	0.112	0.038	0.066	0.088	0.123	0.105
ANN-Spec	0.087	0.088	0.045	NaN	0.155	0.085	0.120
Consensus	0.021	0.113	0.018	0.040	0.040	0.133	0.086
GLAM	0.026	0.038	0.016	NaN	0.046	0.048	0.047
MEME	0.067	0.107	0.043	0.071	0.111	0.139	0.125
MotifSampler	0.060	0.107	0.040	0.067	0.098	0.101	0.100
Weeder	0.086	0.300	0.072	0.152	0.161	0.289	0.225

The reason that weeder performs best with respect to all datasets maybe due to its well designed voting and re-ranking mechanism, while the stand-alone Weeder without voting does not perform well in the previous real dataset experiments. Nevertheless, the results are not very meaningful to offer knowledge to understand motifs, because both the benchmark generation (from poor input data and conservation) and the best performance (based on voting mechanisms without modelling support) need further investigation for their appropriateness for evaluating motif discovery programs.

As a result, the performance of GALF-G on this benchmark, as well as any other algorithms reported in [1], do not offer much knowledge to better understand the supposedly underlying "motifs". On the contrary, many datasets of this benchmark are beyond the common premises of data requirement and conservation concept for (sequence-based) motif discovery and thus it would not be very meaningful to draw any conclusion. It has been indicated that the

Tab. 7: Non-zero performance of cases of GALF-G ($v_{threshold} = 90\%$) on the Tompa et al benchmark, compared with Weeder on nucleotide, binding site levels.

GALF-G

Datasets	nSn	nPPV	nPC	nCC	sSn	sPPV	sASP
dm01g	0.37	1.00	0.37	0.60	0.43	1.00	0.71
dm06r	0.09	0.41	0.08	0.18	0.14	0.50	0.32
hm17g	0.07	1.00	0.07	0.26	0.10	1.00	0.55
mus01r	0.07	0.20	0.06	0.09	0.17	0.50	0.33
mus10g	0.04	0.91	0.04	0.20	0.07	1.00	0.53
mus12m	0.11	1.00	0.11	0.32	0.14	1.00	0.57
yst02g	0.14	0.56	0.13	0.26	0.20	0.50	0.35
yst04r	0.16	0.74	0.15	0.34	0.33	1.00	0.67
yst08r	0.24	0.67	0.22	0.39	0.50	0.64	0.57
yst09g	0.24	0.98	0.24	0.48	0.38	1.00	0.69

Weeder

Datasets	nSn	nPPV	nPC	nCC	sSn	sPPV	sASP
dm01g	0.00	NaN	0.00	NaN	0.00	NaN	NaN
dm06r	0.00	0.00	0.00	-0.02	0.00	0.00	0.00
hm17g	0.61	0.89	0.57	0.73	0.90	0.90	0.90
mus01r	0.00	0.00	0.00	-0.04	0.00	0.00	0.00
mus10g	0.25	0.50	0.20	0.35	0.47	0.50	0.48
mus12m	0.00	NaN	0.00	NaN	0.00	NaN	NaN
yst02g	0.55	0.70	0.44	0.60	1.00	0.71	0.86
yst04r	0.11	0.25	0.09	0.16	0.29	0.33	0.31
yst08r	0.28	0.60	0.24	0.40	0.57	0.62	0.59
yst09g	0.29	0.56	0.24	0.40	0.62	0.57	0.59

improved benchmark [2] is more appropriate to test motif discovery algorithms for meaningful motif, and we have shown the comparison results in the main paper.

Exceptionally unconventional modelling is more likely to be the direction to seek the solutions for the Tompa et al benchmark. In fact, before we can investigate any unconventional models, we need to revisit the biological and practical significance of this benchmark.

5 Parameter Setting for the Improved Eukaryotic Benchmark Experiments

This section corresponds to the running parameters for GALF-G in sub-section: **Experiments: Single Motif Discovery Challenges on Eukaryotic Bench-**

marks of the paper.

The eukaryotic benchmarks [2] are challenging where the signals are weak and only top-scored motifs are output for comparisons. Therefore, it is suggested to remove non-informative repeats before searching [4]. In GALF-G all repeats longer than 6 are masked by setting the corresponding input parameters (see the GALF-G help). It is also more appropriate to employ the ZOOPS (zero or one occurrence per sequence) assumption, as MEME does by default, rather than finding multiple occurrences which is more difficult to handle. We also suggest that a short width range and close to common motif widths ($[8, 12]$) should be used to capture the weak signals. In the experiments for the improved benchmark [2], the fixed width 8, width ranges $[8, 10]$, $[8, 12]$ and $[8, 16]$ were tried. The average performances (nSn, $nPPV$, nPC and nCC) are shown in Table 8. The width ranges in general are significantly better than a single fixed width (8) in all performance measurements, demonstrating the superiority of the generalized model. The ranges gave very close performances (differences < 0.003 in nCC), except that the nCC of $[8, 16]$ dropped to 0.089 in the Markov background datasets. It indicates that for challenging problems with weak conservation of the motifs, it is more appropriate to use small ranges to capture the subtle signals. In the paper we present the GALF-G results with range $[8, 10]$.

Tab. 8: Average performances (nSn, $nPPV$, nPC and nCC) of GALF-G with different width ranges on the algorithm benchmark suite (50 datasets with Markov backgrounds and 50 with real backgrounds)

Widths	Markov				Real			
	nSn	nPPV	nPC	nCC	nSn	nPPV	nPC	nCC
8	0.063	0.104	0.051	0.073	0.068	0.110	0.053	0.079
$[8, 10]$	0.117	0.184	0.102	0.138	0.116	0.156	0.095	0.126
$[8, 12]$	0.116	0.178	0.103	0.135	0.116	0.158	0.098	0.127
$[8, 16]$	0.088	0.114	0.070	0.089	0.136	0.141	0.095	0.127

6 Genetic Algorithms

This section corresponds to the introduction to genetic algorithm (GA) in subsection: **Methods: GALF-G Implementations** of the paper. GALF for motif discovery is shown in the brackets as the illustrative example.

The working mechanisms of the genetic algorithm (GA) are briefly introduced (GALF for motif discovery shown in brackets) as follows. A GA (GALF [5]) maintains a population of candidate solutions, called individuals (a set of TFBS instances R which form a motif PFM Θ, represented by their positions $A = \{p_1, p_2, ...\}$), and performs optimization or search (maximize the fitness f of the motif PFM, such as Information Content IC) iteratively. In each iteration named a generation, part of the individuals are chosen by parent selection, and generate offspring (new individuals) via genetic operators such as mutation

and crossover (randomly changing a TFBS position (p) and mixing two set of TFBSs (A_i, A_j) respectively in GALF). The resulting population is subject to survivor selection based on fitness f (crowding [12] is used in GALF, i.e. keeping the fitter individuals from the pairs of similar parents and offspring), where unfit individuals will be eliminated to maintain a constant population size. The fittest surviving individual(s), towards convergence, e.g. unchanged for a long period, or at the end of all generations, will be output as the final solution(s). The balance between convergence (local filtering) and divergence (crowding) needs to be maintained by various general and problem specific operators for good performance.

7 Extended GALF Operators

This section corresponds to the content of **Extended GALF Operators** from sub-section: **Methods: GALF-G Implementations** of the paper. A small set of experiments are performed on GALF independently to verify the modifications on local filtering.

The original similarity score used in local filtering (LF) in GALF [5], sim, is first re-introduced. The following sub-sections compare the performance of the proposed log likelihood ratio $logp$ (defined in Equation 9 in the paper) used in GALF-G, with sim on the 8 real datasets from [4]. The information of the datasets is summarized in Table 2 in the paper. Muti-point crossover, with crossover points being half of the dimension number of an individual, is employed to achieve similar effect of uniform crossover with less cost.

7.1 The Original sim Score

In the original GALF, the similarity score sim used in local filtering (LF) with the consensus concept is defined as follows:

$$sim(r_i) = \sum_{j=1}^{w} \Theta_{jr_i(j)}, \tag{13}$$

where $r_i(j) \in \Sigma$ is the nucleotide in column j of r_i, $\Theta_{jr_i(j)}$ is the corresponding frequency from the PFM (see the example from Figure 7 in the paper). However, the relation between this heuristic score and the fitness of statistical models is not clear. sim serves as a sum of the continuous similarities, corresponding to the sum of discrete edit distances in consensus-led methods. The similarity score is not directly related to the current probabilistic models, and does not consider the background information. As a result, sim has the risk leading the instances to suboptimal ones. $logp$ is therefore proposed as a better score in the paper.

7.2 Experiment Setting

In order to investigate into the effect of *logp* and the crossover clearly without involving other complicated factors, we examined the performance of GALF alone without any post-processing on the 8 real datasets [4]. The experiments were run with the same parameter setting specified in the paper for GALF [5]. All the experiments were executed on the same Pentium D 3.00 GHz machine with 1GB memory, running Windows XP. The results were averaged on 20 runs of GALF (note that we focus on the average performance of each single run of GALF, whereas in one single execution of GALF-P, GALF is already run 20 times to achieve the best GA results. Thus the experimental results following will look worse than what were presented in [5]).

The 8 datasets chosen for experiments suit the OOPS (one occurrence per sequence) assumption well because each of them has at least OOPS, and 3 have exactly OOPS. GALF is based on the assumption of OOPS as well and IC is used as the fitness, which turns out to eliminate the other modelling difficulty involved, such as adjusting the abundance ratio of the TFBSs. As a result, the fitness (IC) and performance should match well with each other and help better demonstrate the difference between *logp* and *sim*. With this OOPS setting (i.e. the predicted TFBS number is always the same for the same dataset), the performance on the site level is determined only with respect to sTP (true positive). It is suffice to compare the precision $sPPV$, where the rankings of both sSn and sF are the same with $sPPV$.

7.3 Results

We ran GALF using *sim* and *logp* combined with single-point and multi-point crossover for 20 runs and compare their fitness and $sPPV$. *logp* makes more significant contributions than the multi-point crossover. Nevertheless, the results for *logp* with multi-point crossover are generally better (by 0.01 in $sPPV$) than with single-point crossover in most of the cases, where the former is significantly better in MyOD with $sPPV$ 0.84 $\pm$ 0.20 VS 0.75 $\pm$ 0.32 (details not shown). For simplicity of demonstration, only the average results of *sim* with single-point crossover and *logp* with multiple-point crossover are shown in Table 9.

Illustrated in the fitness comparisons in Table 9 left, *logp* with multi-point crossover generally achieves better average fitness and significantly lower standard deviations than *sim* with single-point crossover except in the case of ERE. However, the difference in performance of ERE is small. It is observed that the higher fitness achieved based on *logp* leads to better performance in Table 9 right, especially in the last 4 datasets, where small differences in fitness reflect significant different precisions $sPPV$, showing the search difficulties. In the extreme case of MYOD, which probably contains several motifs with similar fitness, the difference in performance is dramatic while the difference between the optimal motif and the sub-optimal ones in fitness is relatively small.

The results show that with *logp* and multi-point crossover, significantly bet-

ter effectiveness can be achieved, and the standard deviations are lowest, in only a single run of GALF. With this improvement, the performance of GALF is reliable and stable enough to make the meta-convergence framework succeed with relatively fewer runs of GALF.

Tab. 9: The average fitness (left) and the average performance with respect to precision $sPPV$ (right) of 20 GALF runs. sim is associated with single-point crossover while $logp$ is associated with milti-point crossover. ± indicates the standard deviation.

Datasets	Fitness		$sPPV$	
	sim	$logp$	sim	$logp$
CREB	8.29<br>±0.01	8.29<br>±0.03	0.76<br>± 0.01	0.77<br>±0.01
CRP	10.09<br>±0.03	10.12<br>±0.03	0.91<br>±0.04	0.94<br>±0.01
ERE	9.09<br>±0.06	9.13<br>±0.18	0.80<br>±0.03	0.77<br>±0.04
E2F	8.56<br>±0.16	8.77<br>±0.03	0.76<br>±0.04	0.77<br>±0.02
MEF2	7.27<br>±0.23	7.73<br>±0.00	0.39<br>±0.38	1.00<br>±0.00
MYOD	6.70<br>±0.04	6.77<br>±0.02	0.28<br>±0.36	0.84<br>±0.20
SRF	10.25<br>±0.06	10.43<br>±0.03	0.72<br>±0.08	0.93<br>±0.08
TBP	6.14<br>±0.74	7.13<br>±0.06	0.63<br>±0.38	0.91<br>±0.02

8 GALF-G Implementation Details

This section corresponds to the extra details of GALF-G from sub-section: **Methods: GALF-G Implementations: Extended GALF Operators** of the paper.

Refinement for Additional Instances

In GALF-G, because the extended GALF only assumes ZOOPS, to handle general cases probably with several occurrences in a sequence, refinement is applied to add instances based on the fitness f upon convergence or when MAXRUN runs are used up. Generally, if a fixed width is input, instances have to increase f in order to be added, while in the width range case, the threshold of f is relaxed slightly.

To reduce the intensive computation of evaluations on all possible instances, only a number of extra candidates besides those in a slot (represented by its

best individual) I_s are selected according to their log likelihood ratio (Equation 13 with $w_0 = 0$ and $w_{cor} = w_{max}$). They are ranked and only those exceeding the threshold th will be considered. Suppose I_s has a core width w_{cor} and the log likelihood ratio $logp_{avg}$ averaged on every instances based on Equation 13, th is determined with the primary threshold $cutoff$ and the adjustment weight wt related to the width:

$$th = (cutoff - w_{cor} * wt) * logp_{avg}. \tag{14}$$

The $cutoff$ is chosen as 0.9, similar to the cut-off criterion employed in motif matching methods [13] (which aims to provide a recognition rate of at least 90%). $wt = 0.02$ is employed to relax the requirement when w_{cor} is long, in order not to miss significant candidate. In fact, wt is not critical for the performance, since whether a candidate shall be included is controlled in the next step. However, the efficiency slightly decreases with too large wt, though it will not affect much on the performance.

A candidate will be finally added if the new f' satisfies

$$f' > (1 - w_{cor} * ft) * f \tag{15}$$

with similar purpose to the previous, where ft is the relaxation weight. For fixed width cases, $ft = 0$. Otherwise $ft = \max((w_{max} - w_{min})/5, 1) * 0.001$. The intuition is that we only relax a little in order not to include degenerative instances. Although more systematic investigation might be worthwhile, this setting works well in all the experiments.

GALF-G Pseudo-codes

The pseudo-code of the new LF is illustrated in Table 10. The whole extended GALF procedure is shown in Table 11. The pseudo-code of GALF-G is shown in Table 12.

References

[1] Tompa M, Li N, Bailey TL, Church GM, Moor BD, Eskin E, Favorov AV, Frith MC, Fu Y, Kent WJ, Makeev VJ, Mironov AA, Noble WS, Pavesi G, Pesole G, Regnier M, Simonis N, Sinha S, Thijs G, van Helden J, Vandenbogaert M, Weng Z, Workman C, Ye C, , Zhu Z: **Assessing computational tools for the discovery of transcription factor binding sites**. *Nature Biotechnology* 2005, **23**:137–144.

[2] Sandve GK, Abul O, Walseng V, Drablos F: **Improved benchmarks for computational motif discovery**. *BMC Bioinformatics* 2007, **8**:193.

[3] Hu J, Li B, Kihara D: **Limitations and potentials of current motif discovery algorithms**. *Nucleic Acids Res.* 2005, **33**:4899–4913.

Tab. 10: Pseudo-code of the local filtering (LF) operator

```
Input: An individual I with the collapsed SIM A = {p_1, p_2, ..., p_m}
   where p_i is the site, i.e. position, (may be null) for instance r_i;
   m is the sequence number.
LOCAL_FILTERING(I)
{
   Choose a random w' for NORMAL or CONVER (w_max) mode
   Choose the offset w_0 randomly from [1, w_max - w' + 1]
   Sort all the instances by logp(·, w_0, w') and obtain their
   corresponding sequence ranking: Rnk(1), Rnk(2), ... Rnk(m);
   where logp(r_Rnk(1)) ≥ logp(r_Rnk(2))... ≥ logp(r_Rnk(m))
   // logp of a null instance is set to be -∞
   for (k = m; k ≤ 2, k - -)
   {
      if ( mode == NORMAL ) {
         Scan sequence Rnk(k) to get q_Rnk(k) with best logp;
         p_Rnk(k) = q_Rnk(k);
         if (logp(p_Rnk(k)) ≤ logp(p_Rnk(k-1))) Return I;
      }
      if ( mode == CONVER ) {
         if (f(I - {p_Rnk(k)}) > f(I)))    p_Rnk(k) = NULL;
         else    Return I;
      }
   }
}
```


[4] Wei Z, Jensen ST: **GAME: detecting cis-regulatory elements using a genetic algorithm**. *Bioinformatics* 2006, **22**(13):1577–1584.

[5] Chan TM, Leung KS, Lee KH: **TFBS identification based on genetic algorithm with combined representations and adaptive post-processing**. *Bioinformatics* 2008, **24**(3):341–349.

[6] Shaw WM, Burgin R, Howell P: **Performance standards and evaluations in IR test collections: cluster-based retrieval models**. *Inf. Process. Manage.* 1997, **33**:144.

[7] Welch BL: **The generalization of "student's" problem when several different population variances are involved**. *Biometrika* 1947, **34**:28–35.

[8] Hertz G, Stormo G: **Identifying DNA and protein patterns with statistically significant alignments of multiple sequences**. *Bioinformatics* 1999, **15**(7-8):563–577.

[9] Hu J, Yang YD, Kihara D: **EMD: an ensemble algorithm for discovering regulatory motifs in DNA sequences**. *BMC Bioinformatics* 2006, **7**:e342.

[10] Wijaya E, Yiu SM, Son NT, Kanagasabai R, Sung WK: **MotifVoter: a novel ensemble method for fine-grained integration of generic motif finders**. *Bioinformatics* 2008, **24**(20):2288–2295.

Tab. 11: The extended GALF. INTL is the interval of generations to trigger LF. MAXGEN is the maximal number of generations to run and MAXCONVER is the convergence count.

```
for(i=0; i < MAXGEN; i++)
{
  Evaluation on the population;
  NORMAL mode LF on the population every INTL generations;
  Randomly pair the N individuals into N/2 pairs;
  for(each pair of the individuals)
  {
    Uniform crossover and Single-point mutation;
    Evaluation and Selection within the pair;
  }
  C = the best individual;
  if(C stagnates for ≥ 1/4MAXCONVER)
    CONVER mode LF on the population;
  if(C stagnates for ≥ MAXCONVER)   break;
}
Output NUM best individual(s) C[·];
```

Tab. 12: The framework of GALF-G. MAXGEN and MAXRUN are the maximal generations of GALF and maximal times to run GALF, respectively. MAXIND is the convergence count for best individuals from different runs.

```
Initialize K+1 Slot[·] for K motif types and the counters Cnt[·];
Initialize a random population with N individuals;
for(g=0; g < MAXRUN; g++)
{
  Re-initialize the population accordingly;
  Run the extended GALF;
  C[·] = the NUM best individuals output by GALF; //GALF in Table 11
  for(i=0; i < NUM; i++)
  {
    for(j=0; j < K+1; j++)
    {
      if(SimilarityTest(C[i], Slot[j]) is passed)
      {
        Slot[j] = the one with better f between C[i] and Slot[j];
        Cnt[j]++;
        if( Cnt[j] ≥ MAXIND )
          Mark Slot[j] as converged and erase Slot[j];
        break;
      }
    }
    if (C[i] does not suit any existing slot)
    {
      if (An empty slot exists)   Put C[i] to that slot;
      else    C[i] competes with the slot with lowest f;
    }
  }
  if(The K best solutions of the K+1 slots converge)   break;
}
Refinement on Slot[·] and output the best K ones in terms of f.
```

[11] Pavesi G, Mereghetti P, Mauri G, Pesole G: **Weeder web: discovery of transcription factor binding sites in a set of sequences from co-regulated genes**. *Nucleic Acids Res.* 2004, **32**:W199–W203.

[12] Mahfoud SW: **Crowding and preselection revisited**. In *Parallel problem solving from nature 2*, North-Holland 1992:27–36.

[13] Kel AE, Goessling E, Reuter I, Cheremushkin E, Kel-Margoulis OV, Wingender E: **MATCH: a tool for searching transcription factor binding sites in DNA sequences**. *Nucleic Acids Res.* 2003, **31**(13):3576–3579.
